# Impact of the ECM on the Mechanical Memory of Cancer Cells

**DOI:** 10.3390/cells14211707

**Published:** 2025-10-30

**Authors:** Claudia Tanja Mierke

**Affiliations:** Faculty of Physics and Earth System Sciences, Peter Debye Institute of Soft Matter Physics, Biological Physics Division, Leipzig University, 04103 Leipzig, Germany; claudia.mierke@uni-leipzig.de

**Keywords:** environmental mechanical cues, mechanical memory, viscoelasticity, stiffness, epigenetics, mechanobiology, deformability, traction forces

## Abstract

**Highlights:**

**What are the main findings?**

- Mechanical memory in the cell-matrix interactions appears to play a significant role at various levels in the metastatic cascade in cancer.
- The effect of mechanical memory influences the effectiveness of therapeutic approaches in cancer.

**What is the implication of the main finding?**

- Knowledge of mechanical memory can lead to improved tumor therapy by combining inhibitors of the altered mechanical properties of the tumor matrix, such as increased stiffness, with established drugs that can better reach the site of action.
- Common mechanical memory mechanisms of different types of cancer point to a universal phenomenon and as such need to be assessed in a dynamic manner to precisely predict the individual malignant potency of tumors.

**Abstract:**

Besides genomic and proteomic analyses of bulk and individual cancer cells, cancer research focuses on the mechanical analysis of cancers, such as cancer cells. Throughout the oncogenic evolution of cancer, mechanical inputs are stored as epigenetic memory, which ensures versatile coding of malignant characteristics and a quicker response to external environmental influences in comparison to solely mutation-based clonal evolutionary mechanisms. Cancer’s mechanical memory is a proposed mechanism for how complex details such as metastatic phenotypes, treatment resistance, and the interaction of cancers with their environment could be stored at multiple levels. The mechanism appears to be similar to the formation of memories in the brain and immune system like epigenetic alterations in individual cells and scattered state changes in groups of cells. Carcinogenesis could therefore be the outcome of physiological multistage feedback mechanisms triggered by specific heritable oncogenic alterations, resulting in a tumor-specific disruption of the integration of the target site/tissue into the overall organism. This review highlights and discusses the impact of the ECM on cancer cells’ mechanical memory during their metastatic spread. Additionally, it demonstrates how the emergence of a mechanical memory of cancer can give rise to new degrees of individuality within the host organism, and a connection to the cancer entity is established by discussing a connection to the metastasis cascade. The aim is to identify common mechanical memory mechanisms of different types of cancer. Finally, it is emphasized that efforts to identify the malignant potency of tumors should go way beyond sequencing approaches and include a functional diagnosis of cancer physiology and a dynamic mechanical assessment of cancer cells.

## 1. Introduction to Mechanical Memory

The physical migration to and invasion of cancer cells of targeted tissue sides during cancer metastasis represents a clear sign of malignant cancer progression that often leads to cancer-related deaths. Cancer cell spreading has been extensively explored based on genetic [1,2], proteomic [3,4] and epigenetic cues [5,6,7,8,9]. However, the metastatic spread of cancer cells is still not fully understood. Thus, the current focus of epigenetic studies on cancer progression has been broadened to mechanical signals [9,10,11,12,13]. Cancer malignant progression compromises mechanical cues of the extracellular matrix (ECM) microenvironment, such as stiffness/softness and viscoelasticity, intercellular tension inside a solid tumor, contractility-driven stress evoked by tumor stromal cells [14], interstitial fluid pressure (IFP) [15], the direct interplay of cancer cells with endothelial cells or pericytes of the vascular system during their possible transmigration of the endothelial vessel lining [16,17], resistance toward blood flow forces, and the physical characteristics at the targeted tissue niche.

This review discusses and clarifies the significance of the mechanical signals to which cancer cells are exposed, as well as their responsiveness in each step in the metastatic cascade. It is also demonstrated how cancer cells can remember these mechanical stimuli. For example, stiffness-induced mechanical memory mechanisms are discussed, whereby the role of structural alterations in the cell’s cytoskeleton, the activity of transcription factors and epigenetic effects are highlighted. Moreover, the focus is placed on how mechanical memory affects cancer cells’ metastatic spread. The influence of mechanical memory on the various steps of metastasis has been proposed and discussed. The universal applicability of mechanical memory enabled by the ECM to various cancer cell types is debated. Finally, the influence of mechanical memory on future directions is also envisioned, with a special emphasis on biomechanical changes.

## 2. The Amount of Mechanical Memory Is Impacted by the Intensity and Length of the Mechanical Stress

**Figure 1:**
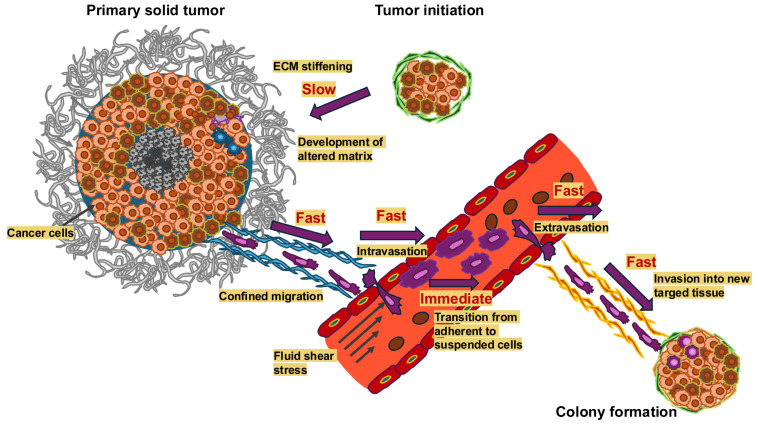
Mechanical alterations take place on vastly different timescales in the course of cancer metastasis. Dynamic alterations in the mechanical microenvironment throughout cancer metastasis arise with varying temporal scales. Important processes like ECM strengthening take a long time, from months to years, unlike fast processes like restricted migration and invasion into new targeted tissue, intravasation (usually 2 to 24 h) and almost fast processes, such as extravasation (typically 24 h to three days) and immediate processes like the transition from adhesion to suspension when cancer cells enter the circulatory system. The longer a mechanical signal lasts, the more probable it is that it serves as mechanical memory.

It has been established that cancer cells constantly sense and adjust to mechanical characteristics of the microenvironment, which impacts their functionality, such as through motility and invasiveness, as well as their metastatic capacity [18,19]. The timescales over which the mechanical signals change in the various steps of the metastasis cascade are depicted in Figure 1. In addition, the exposure time of cancer cells to these mechanical cues seems to also be a critical factor. Beyond that, there is limited knowledge about how cellular adjustments from past physical surroundings are preserved or how cells store mechanical memory on long (several days or weeks) or short (a few days) timescales.

### 2.1. What Are the Timescales of Mechanical Memory?

**Figure 2:**
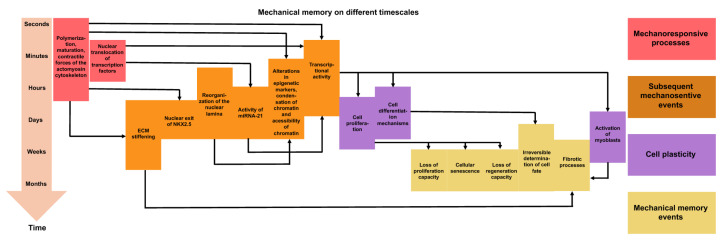
Mechanical memory can manifest itself over very different periods of time. Overview of the processes that occur at the various phases of mechanical activation, mechanotransduction, and mechanical memory. The arrows point to the subsequent consequences. The different colors indicate the specific type of processes. The mechanoresponsive events that are directly triggered through mechanical stress are illustrated in red. The downstream mechanosensitive events are reversible under transient mechanical stress and are highlighted in orange. The cell plasticity events or instances of targeted phenotypic alterations are marked in purple. Mechanical memory events, which occur (in part) as a result of persistent mechanical stress, are shown in yellow.

Current knowledge about mechanical memory can be summarized in two points [20]. First, the memory of retrograde stiffness is dose-dependent and grows with the priming time to the point where the effects begin to take on irreversibility. In many short-term mechanical priming events, the processes can be reversed, and the reversibility of different mechanical priming mechanisms has recently been extensively reviewed in [21]. The critical time-point prior to irreversibility probably relies heavily on the state of the cell such as its mechanosensitivity, differentiation capacity, and the cell cycle phase, as well as on the time-dependent signaling paths that become active when specific differentiation signals are received. There seems to be no systematic investigation yet into the extent to which mechanical memory relies on the magnitude of the stiffness mismatch, although this has been investigated on a theoretical basis [22]. It can be assumed that this phenomenon is nonlinear, as the mechanical stress on cells caused by the traction forces between cells and the extracellular matrix grows nonlinearly with the stiffness of the substrate [23]. Traction forces are defined as tangential forces exerted by cells either to the ECM or the underlying substrate [24]. The second point of convergence is that there are a number of effectors of mechanical memory whose activity varies depending on cell types and the nature of mechanical stress. The characteristic time constants relevant to mechanical memory are the period of the mechanical priming phase and the stability of proteins and epigenetic determinants, as well as the combined reaction time of the feedback circuits between the cytoskeleton and transcription (Figure 2). In current computer-based investigations, these factors on their own were all that was necessary for mechanical memory [25,26].

### 2.2. Physical Priming Affects Mechanical Memory

The insight into mechanical memory lies in gaining an appreciation of how physical priming effects within a specific microenvironment will influence cellular function and destiny within a different, subsequent microenvironment [27,28,29]. In certain cases, when the amount of time spent in physical priming is somewhat restricted, the mechanical memory can be retained for a short period of time, such as 1 to 3 days, and it has no effect on the long-term behavior of the cells [30]. In contrary, the accumulated memory of this environment diminishes cellular plasticity when the duration of mechanical environment exposition exceeds a critical limit, leading to the reprogramming of cells into a condition with a sustained phenotype through the regulation of focal adhesion assembly and architecture [28,31,32]. Thereby, the long term mechanical memory keeper MicroRNA-21 has been identified [28]. On some occasions, this reprogrammed mode can be abnormal and exacerbate conditions like fibrosis [28,31,33], affect cell performance during cancer metastasis [34], or impair regenerative tissue processes [28,29,30]. Nonetheless, the mechanisms of mechanical memory are not yet fully comprehended, and it is crucial to explore how to perturb maladaptive mechanical memory for translational application purposes.

As the stimulation of mechanoreceptors, such as integrins, requires to be above a certain threshold [35], it seems to be likely that there also exists a threshold for mechanical memory stimulation. This would be necessary to avoid unnecessary information storage and to save only relevant changes. Thus, on short priming times the cells adapt temporarily to substrate stiffness, but mechanical signaling cannot increase reinforcement (transcriptional activity) sufficiently to generate mechanical memory. Consequently, there is no memory generated. Apart from exceeding a specific stiffness limit for generation of mechanical memory, the mechanical dosing plays a role as it has been previous demonstrated in human mesenchymal stem cells (hMSCs). Typically, many of the experimental investigations have centered on the influence of substrate stiffness and the impact of mechanotransduction on hMSCs, specifically how hMSCs perceive and assimilate mechanical signals from their rigid environment, which subsequently dictate their cellular fate [31,32,33]. For instance, hMSCs can memorize previous mechanical surroundings, which may impact long-term decision-making regarding cell fate or regenerative ability after transplantation [28,34]. It is assumed that cancer cells experience a similar change in their mechanical cues and memorize previous mechanical environments when they undertake the metastatic journey. This mechanical memory is featured by an irreversible nuclear homing of the co-transcription factor YAP and relies on the duration of hMSC cultivation on stiff substrates, which is referred to as mechanical dosing. The mechanical dosing may also play a role in cancer cells. In the past, the event of stress/strain stiffening has been described for cancer cells. The stiffening of cancer cells under stress has been delineated by a behavior in which the internal architecture of the cells, such as their actin cytoskeleton, becomes less flexible and stiffer under elevated mechanical stress [17,36,37,38]. Previously, it has still been interpretated as not fully relaxed cells that are mechanically stimulated several times. This strain stiffening enables these cells to both reshape themselves and squeeze through tight spaces to penetrate tissue, as well as generate force and adhere better to the ECM during invasion and metastatic spread. This strain stiffening process involves a complicated, nonlinear mechanical reaction in which the cell adjusts its stiffness depending on the strain to adapt to mechanical constraints. This stiffening is a kind of mechanoadaptation, which implies that the cell alters its mechanical characteristics to deal more effectively with the stresses and strains experienced in invasion, such as migration through constrained tissue and the ECM scaffold. Assuming these mechanical adjustments persist, they can be considered a form of mechanical memory. A brief cell cultivation period on stiff substrates (short-term mechanical dosage) leads to reversible mechanical memory, in which YAP can relocate from the cell nucleus to the cytoplasm with nominal modifications in gene expression. In contrast, prolongation of the mechanical dosing leads to irreversible mechanical memory, where YAP remains in the cell nucleus and induces significant alterations in gene expression [28,32,34,39,40]. It is widely accepted that YAP and some other transcription factors can modulate gene expression. However, it is common knowledge that epigenomics can likewise modulate gene expression, which points to its involvement in mechanical memory [41,42,43,44,45,46]. At intermediate priming times, the mechanical signaling increases reinforcement but leads to only temporary memory. The model predicts a continuous range of memory persistence times, from significantly shorter than priming time to longer than priming time, which relies on the time and stiffness of the priming phase. At long priming times, the reinforcement from mechanical signaling becomes strong enough to result in permanent memory in which the cell phenotype persists even if the substrate is switched back.

It has been discussed that chromatin remodeling, such as the architecture of chromatin, may serve as a mechanical memory keeper [29]. Chromatin rearrangement predominantly controls gene expression via epigenetic modifications involving acetylation, methylation, and phosphorylation at the amino-terminal ends of nucleosomal histones (see also Figure 1) [47,48,49,50]. The acetylation scene is super dynamic and is controlled by two types of enzymes, such as histone acetyltransferases (HATs) and histone deacetylases (HDACs). The acetylation of histones through HATs results in the expansion of chromatin, which facilitates gene expression [47,48,49,50]. As an alternative, the deacetylation of histones by HDACs leads to chromatin condensation and suppression of gene expression. HDACs and HAT1 have been demonstrated to fulfill tasks in reversible and irreversible remodeling of chromatin [29].

### 2.3. Mechanical Cues Evoked by Culture Conditions Impact the Response to Medication

Previous investigations have demonstrated that mechanical stimuli can impact the epigenomics of hMSCs [27,51,52,53,54,55]. For instance, it was revealed that in MSC-colonized scaffolds exposed to 10 percent tensile stretch, the condensation of chromatin was enhanced by about 80 percent in comparison to unstressed controls [51]. Moreover, the results indicated higher and sustained rates of condensation following multiple exposures of the scaffolds to stress, indicating that previous stress had affected the cell nucleus [27]. Similarly, it has been demonstrated that topology impacts epigenomics, as MSCs grown on 10 µm grooved polydimethylsiloxane (PDMS) surfaces exhibited increased histone 3 acetylation levels and reduced nuclear HDAC activity when compared to cells grown on smooth surfaces [56]. Moreover, they showed that the adult fibroblasts were able to undergo EMT on these grooved substrates. Finally, it has been found that the expansion of MSCs on tissue culture plastic not only reduces secretory characteristics and multipotency, but also causes some localized genetic and epigenetic alterations, such as DNA methylation increasing over time [57]. Overall, these data imply that chromatin reorganization and the epigenetic map probably serve as key drivers in how hMSCs incorporate physical signals from their environment through time. Nevertheless, it is still uncertain whether these chromatin alterations can be reversed and what part integrated exposition to stiff microenvironments over time, such as mechanical dosing, can take in controlling these alterations. Therefore, the epigenomic and chromatin rearrangement that arises in expanding hMSCs hinges on the history of the cultivation and cumulative alterations in chromatin architecture over time result in a mechanical memory of the cell. Altogether, there seems to be a nonlinear coupling between mechanical signaling and transcriptional evolution that can explain the general features of mechanical memory. Nevertheless, further experimental studies are required to extend this model and include mechanistic details, this model may represent an important foundation for experimental studies and cell-based therapies that aim to engineer cell dynamics based on microenvironment mechanics.

The question arises as to how typical standard culture methods such as spheroids or feeder layers perform when modeling the various conditions of mechanical input. Spheroids constitute 3D cell clusters that are self-assembling and more closely resemble solid tissue compared to 2D tissue cultures. They enhance interaction between neighboring cells and between cells and the matrix, providing a physicochemical environment analogous to that found in living organisms. The spheroids retain their intrinsic phenotypic characteristics and promote the expression of stem cell markers. In addition, cytokines, chemokines, and angiogenic factors are secreted by spheroids, thereby increasing cell viability and proliferation [58]. An advancement is the possibility to include particles in spheroid cultures to govern mechanotransductional mechanisms inside the spheroid and increase viability and proliferation [59]. Modeling of the mechanical environment of solid tumors, incorporating the implications of cell–cell and cell-ECM interactions, is highly suitable for mechanical input modeling. Cells within spheroids are subjected to shear stress and compression, with mechanical characteristics, such as mechanical forces varying depending on cell type and size. Larger spheroids (>500 µm) generate metabolic gradients (hypoxia, dormancy, proliferation) that are reminiscent of those observed in tumors and are impacted through mechanical factors, which is another advantage of these models. An advantage of them is their resistance to drugs. In particular, their 3D structure and mechanical characteristics encourage drug resistance resembling that of solid tumors, which renders them useful for drug screening. A disadvantage is that they exhibit a diffusion gradient with increasing size of the spheroids and a deficiency of nutrients in the center of the spheroids [58]. Another limitation is that, although they represent a considerable advancement over 2D models, they simply do not possess the complexity and engineering framework of organoids, and visualizing their internal structure can be complicated. Feeder layer cultures consist of a layer of cells (the feeder layer) that serves as a substrate for the growth of other cells. Feeder layer models are of limited suitability for mechanical input modeling, as they primarily offer a 2D interface. Feeder layers transmit mechanical forces predominantly in a 2D plane. They can be coupled with other methodologies, such as 3D culture systems, to achieve a more advanced mechanical microenvironment. Feeder layers are somewhat limited, as they mainly model mechanical impacts in a 2D plane and may not completely reflect the intricate forces in 3D tissues that cells are exposed to in a real tissue microenvironment. Special culture systems are required to precisely regulate and accurately measure mechanical forces in 2D or 3D. In conclusion, spheroid and feeder layer methods provide distinct opportunities for applying mechanical influences, with spheroids being more suitable for modeling mechanical in vivo environments. Both are preferred as cell culture systems over simple 2D cell culture on plastic dishes. In this context, it can be cautiously said that standard monolayers on plastic are not very significant. The question arises as to which culture conditions (even xenografts, while inappropriate for large-scale trials) are more suitable than standard monolayer cultures for predicting the reaction to drugs in the context of drug screening. This question can only be answered in part, as there is currently insufficient literature on cancer drugs and mechanical effects, and further efforts are needed. Dynamic suspension cultures such as spinner cultures and rotary wall vessels exert mechanical forces by fluid flow and rotation, which can also enhance nutrient and oxygen delivery. Microfluidic devices enable accurate regulation of mechanical forces like fluid flow and shear stress and can be utilized to explore cell confinement and cancer progression. Specific culture or bioprinting techniques or lap-on-a-chip techniques can be used to regulate the mechanical stimulation [60] to create mechanical memory. Bioprinting is a type of technique that involves using hydrogels and other substances to build intricate 3D structures that permit accurate positioning of spheroids and the exertion of defined mechanical forces. Scaffolds can serve as materials that can be used to produce artificial tissue with controlled mechanical characteristics, enabling investigation into how mechanical forces impact cell performance. All these culture techniques may help to improve the in vitro high-throughput anti-cancer drug screening by also covering the aspect of mechanical cues. However, there is still a lot effort needed to develop a screening platform that is reliable. Considerable progress has been made in the field of mechanosensory and mechanotransduction research, leading to the identification of mechanical memory and its relevance to the malignant progression of tumors.

## 3. Mechanosensing, Mechanotransduction and Mechanical Memory at the ECM-Cell Interface

**Figure 3:**
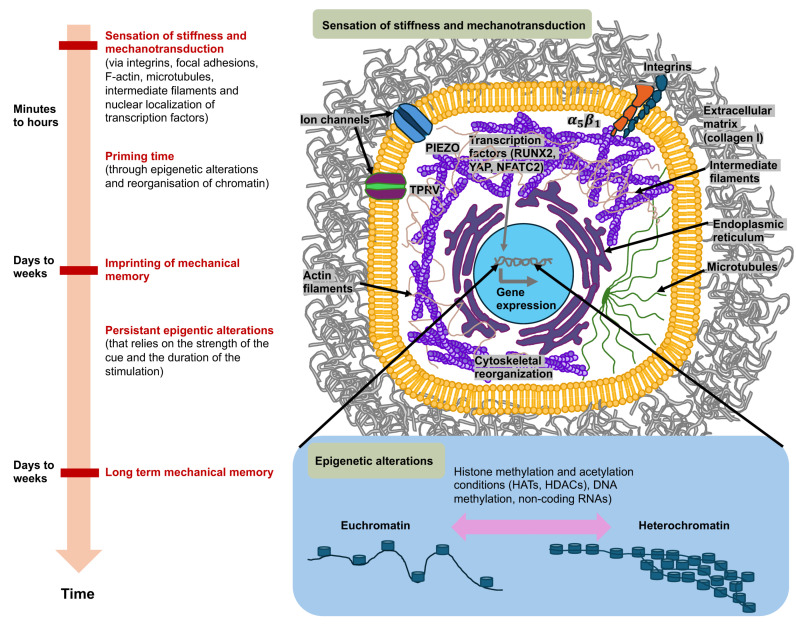
Mechanosensory systems, mechanotransduction, and mechanical memory of cancer cells are characterized by ongoing epigenetic modifications in the most extensively studied context of matrix stiffness. Cancer cells perceive high matrix stiffness through integrin activation, which encourages cytoskeletal reorganization, which then facilitates the translocation of mechanotransducing transcription factors, runt-related transcription factor 2 (RUNX2), and yes-associated protein (YAP), to the nucleus. These mechanosensory and mechanotransduction mechanisms arise in minutes to hours after encountering a rigid matrix. In the long term, mechanotransduction can result in epigenetic alterations, which include histone modifications by histone acetyltransferases (HATs) and histone deacetylases (HDACs), methylation of DNA (typically leads to gene silencing), and expression of non-coding RNAs, which initiate transcriptional gene silencing. When the level of mechanical stress and time of priming are severe enough (days to weeks), phenotypic adjustments are assumed to be preserved by the mechanical memory of cancer cells, which is enacted through sustained epigenetic alterations. Mechanical memory mechanisms appear to account for a key part of the metastasis pathway in that they directly couple the biophysical adaptations of cancer cells inherited from the primary cancer with the adaptations that propel the advancement of the disease to secondary cancer locations.

Cancer cells perceive mechanical signals that are generated either by direct forces, including tensile, compressive, and shear forces, or indirectly by alterations in the structure and mechanical features of the surrounding microenvironment, for instance, stiffness (elastic modulus) or the architecture of the ECM [5,36,61,62]. The sensing of mechanical forces involves mechanosensors, such as ion channels like PIEZO1 and TRPV4, integrins, that subsequently trigger the activation of molecular effectors in specific signaling routes [63] that can lead to modifications in gene expression and cell function [5,6,64,65]. This phenomenon is referred to as mechanotransduction [63]. For instance, these mechanical cues can facilitate a dynamic reorganization of the cell’s cytoskeleton, which can elevate either its stiffness or its deformability (softness), as it is reviewed in [36]. Through this reorganization of the cytoskeleton, it is transferred from a stiff or ordered state to an irregular or compliant network that can translocate from a cytoplasmic side to a perinuclear side during force exertion [66]. Thus, cells react to mechanical signals through alterations in their internal cytoskeleton and by triggering biophysical alterations to the ECM, comprising its composition, crosslinks, geometry, mechanical properties, and topology [17,67]. Their phenotypic adaptivity is important for the ability to survive and function under altered mechanical environments [15,68,69]. Intriguingly, cells not only perceive mechanical stimuli, but can also remember them after they are no more applied. This kind of long-term mechanotransduction [70] is referred to as “mechanical memory” and could have significant consequences for tissue development, differentiation, and disease progression, such as cancer metastasis, heterogeneity of the cancer and the responsiveness to therapeutic treatment (Figure 3).

In cancer cells, the activation of PIEZO1 play a prominent role in the malignant progression of cancer. PIEZO1 constitutes a homotrimeric complex comprising a single cation-permeable pore [71,72]. Specifically, PIEZO1 in mice has a three-bladed, propeller-like architecture with three long intracellular arms that rotate together like a lever in reaction to mechanical force [73,74]. PIEZO1 is subject to reversible, flattening deformation upon exertion of force [75], which efficiently opens the pivotal pore for cation-selective permeation [75,76]. The PIEZO1 channel is mechanically driven through the actin cytoskeleton via a cadherin-ß-catenin-vinculin complex, indicating that a model considering forces originating from filaments can be combined with a model considering forces originating from lipids [77]. However, further research is required, particularly at the molecular and structural levels, to ascertain precisely how such forces are transmitted. Piezo channels also feature an inactivation gate, which is susceptible to membrane voltage and inhibits additional mechanical excitation until the channels are reset through outward permeation [78]. Apart from cancer cell mechanotransduction, there is also a judge impact of PIEZO1 signaling in immune cells, such as natural killer (NK) cells. The reactivity of NK cells is controlled by the stiffness of cancer cells. The killing efficacy of NK cells in 3D is compromised towards softened cancer cells, while it is increased towards stiffened cancer cells [79]. In T cells, it has been found that PIEZO1-driven mechanosensing of fluid shear stress enhances the activation of T cells [80]. These results indicate that the mechanophenotype of cancer cells is important for their survival, especially during the malignant progression of cancer.

Increased interactions between cancer cells and the ECM can therefore alter the mechanical response of cells through an interlinked hub of mechanochemical systems, including adhesion receptors like integrins, intracellular focal adhesions like talin-1, vinculin, focal adhesion kinase (FAK) and Crk-associated substrate (p130Cas or synonymously referred to as breast cancer anti-estrogen resistance 1 (BCAR1)), cytoskeletal networks such as actin, microtubules, and intermediate filaments, and molecular motors like myosin [17,81]. Talin-1 comprises at its N-terminus a FERM domain harboring four globular segments (F0 to F3), a disorganized connecting region, and a C-terminal rod consisting of 13 four- and five-helix bundles (R1 to R13) [82] which ends in a single α-helix that imparts homodimerization and therefore is designated as the dimerization domain [83].

The most extensively examined activation pathway of talin-1 relies on the small GTPase Rap1, which has an effector, Rap1-GTP-interacting adaptor molecule (RIAM), that connects with talin in R2-R3 in a direct, high affinity manner. There are additional RIAM binding sites in talin-1 that are located in R8 and R11 [82,84]. Rap1 can also bind directly to talin F0 [85,86]. Rap1/RIAM targets talin-1 to the plasma membrane [87,88] counteracts the autoinhibition of talin to encourage the engagement with integrin and actin. A minimum of 9 of the 13 rod domains harbor cryptic vinculin binding sites (VBSs) [89], which are uncovered through mechanical force, enabling vinculin engagement and strengthening of the adhesion. A head (F3) and R9 interaction autoinhibits talin-1, which needs to be liberated for actin and integrin attachment and its subsequent commitment to focal adhesions [90,91,92,93]. The interplay of talin-1 with the negatively charged phosphatidylinositol-4,5-bisphosphate of the inner face of the plasma membrane also supports the activation of talin-1 and its interaction with the plasma membrane [94,95,96]. Talin-1 couples integrins to F-actin partly through the linkage of its N-terminal FERM domain to integrins’ cytoplasmic domains and partly through two locations of its C-terminal flexible rod domain that tethers to F-actin [97]. By sensing the extracellular stiffness, focal adhesion proteins unfold in specific regions/domains, such as talin-1 in its α helix bundles, named R1 to R13, that carry out reversible spring-like behavior (unfolding under tension and refolding when tension removed) [98]. Upon the unfolding of talin-1 vinculin binding is increased [99,100], which consequently rises the tension of talin-1 and strengthens focal adhesions that displays its mechanosensory functional role [83]. The latter finding indicates that talin-1 serves as a signaling hub for mechanotransduction. When vinculin is silenced in a migrating cell, the active localization of mitochondria at the cell front is disrupted, correlating with a reduction in migration velocity after leaving the matrix confinement, which implies a disturbance in cellular mechanical memory [101]. These mechanochemical systems trigger mechanotransduction routes in cancer via the activation of ERK signaling, rearrangement of the cytoskeleton, Rho-GTPase-driven cellular contractility, and cluster formation of integrins [67]. Consequently, the dynamic interaction of cancer cells and their ECM surroundings can impact the shape of the tissue. For example, aberrations in cell-to-cell and cell-to-ECM adhesion, as well as in cytoskeletal reorganization, cause the cancer cell to adopt an invasive shape that can penetrate the ECM, setting off the cascade of metastasis [102]. It has been shown that elevated ECM stiffness can upregulate lamellipodin expression [103]. This is conveyed through an integrin-dependent FAK-Cas-Rac signaling route and assists the stiffness-driven induction of lamellipodin. In this process, FAK and p130Cas transfer the stiffness of the surrounding matrix into internal stiffness of cells [104], which represents a potential mechanism for storing mechanical memories. The protrusion of lamellipodia and filopodia on the plasma membrane is decisive for different mechanisms, such as the migration of individual, mesenchymal or cancer cells [105]. The actin-binding protein lamellipodin is hypothesized to have a pivotal function in lamellipodium protrusion by transporting Ena/VASP proteins to the trailing plus ends of growing actin filaments and engaging with the WAVE regulatory complex, which activates the Arp2/3 complex, at the anterior margin. By counteracting abundant, disorganized retraction and membrane wave formation (ruffling), lamellipodin improves protrusion generation and the development of nascent adhesions [106]. Mechanistically, overexpression of lamellipodin elevated stiffness-induced expression of cell cycle protein D1 [107], while knockdown of lamellipodin diminished it, and triggered entry into the S phase and cell proliferation, pointing to a mechanosensitive cell cycle and subsequently triggering intracellular stiffness. The function of p130Cas as a molecular switch seems to be differently compared to talin-1. First of all, p130Cas has a role as an adaptor protein that acts before talin-1 comes into play at focal adhesions. The substrate p130Cas of the Src family kinase (SFK) has been seen to be phosphorylated and coupled with its Crk/CrkL effectors in clusters that serve as precursors for focal adhesions [108,109]. p130Cas is amongst the primary substrates for integrin-stimulated tyrosine phosphorylation [110,111,112]. The initial phospho-p130Cas clusters comprise integrin β1 that has an inactive, bent, closed state. On the molecular scale, p130Cas harbors an N-terminal SH3 domain, a four-helix bundle, and a C-terminal FAT domain, which are physically spaced apart from one another through unstructured regions and an SFK-SH3/SH2 binding site. The SH3 and FAT domains of p130Cas ensure its localization at focal adhesions [113,114], which can tether other focal adhesion proteins, such as vinculin, FAK, and paxillin as demonstrated in vitro assays [115,116,117]. In a subsequent step, the amount of phospho-p130Cas and total p130Cas is reduced due to the activation of integrin β1 key focal adhesion proteins, including vinculin, talin, kindlin, and paxillin, are enlisted. The formation of p130Cas clusters involves p130Cas, Crk/CrkL, SFKs, and Rac1, whereas vinculin is not necessary. Rac1 ensures positive coupling to p130Cas via reactive oxygen that is compensated by negative ubiquitin-proteasome system feedback. These findings suggest a two-stage model for focal adhesion formation, wherein clusters of phospho-p130Cas, effectors, and inactive integrin β1 propagate via positive feedback before activating integrins and enlisting key focal adhesion proteins. p130Cas and SFKs activate one another, with p130Cas tethering to SFKs and activating them, and SFKs phosphorylating p130Cas at up to 15 repeated YxxP motifs in the substrate domain (SD) sandwiched between the SH3 domain and the quadruple helix bundle [110,118]. The p130Cas SD undergoes fast phosphorylation upon cell adhesion [119,120,121]. The phosphorylation of p130Cas in its SD domain is stretch-dependent [108]. Thereby, p130Cas is able to convert force into a biochemical cue via the extension of the SD domain that leads to a priming to phosphorylation. This mechanical mechanism appears to be of a general nature in controlling intracellular signal transduction events. Phosphorylated pYxxP motifs of p130Cas can attach to specific SH2 domain proteins, such as the paralogs Crk and CrkL. Crk/CrkL, meanwhile, are able to attach to and stimulate several proteins, such as the guanine nucleotide exchange factor (GEF) Rac1 DOCK180 [110,122,123,124,125,126]. After DOCK180 driven activation of Rac1, actin polymerization and lamellipodial protrusion is encouraged by Rac-1 via the WAVE/Arp2/3 complex, and triggers the formation of focal adhesion complexes according to undisclosed principles [127,128,129]. The role of mechanical signals from the TME in terms of their influence on the mechanical memory of cancer cells is discussed below.

## 4. Role of Microenvironmental Mechanical Cues on Mechanical Memory of Various Cancer Cell Types

**Figure 4:**
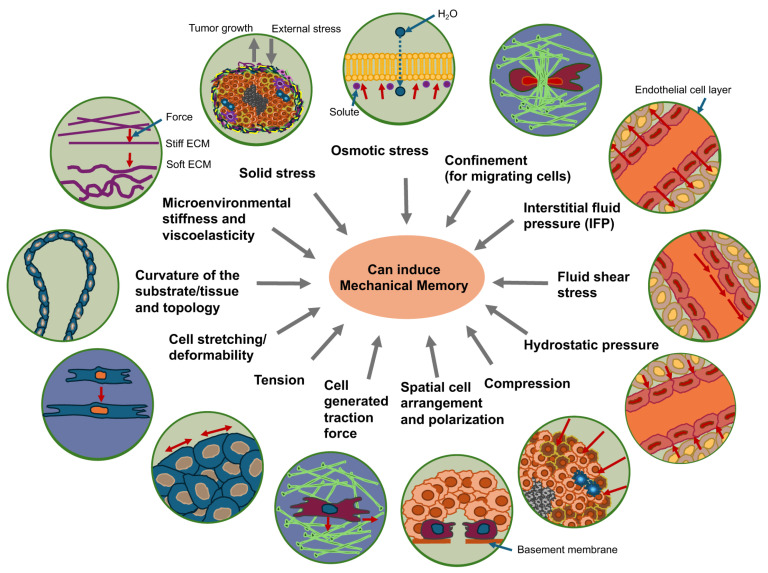
Mechanical cues can trigger the mechanical memory function in cancer cells. Environmental stiffness and viscoelasticity, curvature and topology of the substrate, solid stress, osmotic stress, confinement of matrix migrating cells, cell stretching/deformability, cell generated traction forces, spatial arrangement and polarization, tension, fluid shear stress, hydrostatic pressure, interstitial fluid pressure (IFP) and compression can all trigger the induction of mechanical memory in cancer cells.

The most important question is what kind of mechanical signals can trigger mechanical memory in cancer cells that impacts their functional performance. It has been proven multiple times that the mechanical stiffness of the surrounding matrix can trigger a process of mechanical memory in cells (Figure 4). In addition to matrix stiffness of cancer cell surroundings, mechanical memory can be induced by cellular confinement affecting cancer cell migration, compressive stress that can lead via direct mechanotransduction to stretching/deformation of cells, cytoskeletal remodeling, altered gene expression, proliferation, migration/invasion or other effects via autocrine and paracrine signaling [130,131,132,133,134,135,136,137], fluid shear stress for cancer cells within the blood vessels, interstitial fluid pressure (IFP), hydrostatic pressure, and solid stress due to tumor growth and the reaction of tumor host tissue toward growth-dependent tumor extension (Figure 4).

The localization of mechanosensitive ion channels on the cell surface often enables them to be the first molecules to perceive exterior physical signals and initiate intracellular biochemical cascades in a mechanism referred to as mechanotransduction [138,139,140]. Mechanosensitive ion channels ease the influx of Ca^2+^ into cells by a mechanism referred to as channel gating, enabling the perception of intricate physical signals and alterations inside the microenvironment. This process necessitates a specific threshold tension of the membrane to trigger a conformational switch from the closed to the open condition. The gating process may also rely on transmembrane voltage that is controlled through ion channels [141,142]. Mechanosensitive gating mechanisms can be roughly divided into two types. The first is direct mechanosensory perception, which is governed by physical modifications in the cell membrane, and the second is indirect mechanosensory perception, which is governed by intracellular signaling processes [138,139,143].

Compressive forces inside the primary tumor, fluid shear stress inside the blood vessels, and other mechanical cues that trigger local alterations in cell membrane curvature, composition, and tension can directly open mechanosensitive ion channels like Piezo1/2, Transient Receptor Potential Cation Channel Subfamily C Member 1 (TRPC1), and Transient Receptor Potential Cation Channel Subfamily V Member 4 (TRPV4) [143]. As an alternative, mechanosensitive molecules on the plasma membrane can indirectly induce the activation of mechanosensitive ion channels by a multi-step process that requires participation of a number of intermediate signaling molecules. This mechanism is usually based on the capacity of G protein-coupled receptors to sense alterations in the extracellular environment and induce a conformational switch in mechanosensitive ion channels like the transient receptor potential cation channel subfamily M member 7 (TRPM7) and TRPV4 [143]. Self-generated forces resulting from actomyosin contractility can also cause the activation of mechanosensitive ion channels within migrating cells [144]. For instance, tension in actin cytoskeletal scaffolds and increased signal transmission via integrins in cell-matrix interactions and cadherins in cell–cell interactions can indirectly impact the regulation of mechanosensitive ion channels [143]. In addition, the viscoelasticity of the microenvironment, microenvironmental matrix curvature and matrix topology can trigger mechanical memory as well as cell deformation via cell-stretching. Cell tension, cell generated traction force and the spatial arrangement and polarization of cells can all contribute to mechanical memory (Figure 4).

Surface curvature and matrix stiffness are two distinct concepts, though they can be related. Surface curvature refers to the physical shape of the matrix surface, while matrix stiffness is a material property that describes its resistance to deformation. For example, a cell on a stiff matrix might develop a more curved or smooth surface, while a cell on a soft matrix might develop a wrinkled surface. Stiffness can influence curvature: The stiffness of the matrix can influence the curvature of structures within or on it. For example, a cell’s nucleus might have a smooth, curved surface when on a stiff matrix but a wrinkled surface on a soft one. Surface curvature is directly related to matrix surface tension through the pressure jump across an interface, as described by the Young-Laplace equation, which connects surface tension γ and curvature κ to the pressure jump ΔP across a spherical interface: ΔP = 2γ/R, where R is the radius of curvature. The curvature determines the magnitude of the surface tension force, with more curved surfaces exhibiting higher stress.

How do these physical parameters influence the interfacial tension between the cell collective and matrix scaffold? Surface curvature affects interfacial tension by stretching tissue on convex surfaces and contracting it on concave surfaces, which can modify local cell density and trigger tissue movements. The surface tension of the matrix, which functions as a stretched elastic membrane, generates pressure that can propel the collective migration of cells [145]. These forces are coordinated through cell–cell and cell-matrix interactions to jointly control the shape, flow, and growth of the tissue. How can the surface curvature affect the interfacial tension? Firstly, the surface curvature impacts cell density. For instance, concave areas of the matrix decrease the surface area, resulting in an augmentation of cell density and crowding [146]. By contrast, convex areas enlarge the surface area, thereby reducing cell density and spreading the tissue. Secondly, surface curvature encourages pressure-based movements. In fact, the development of a meniscus, which is a curved interface between tissue and matrix, can generate hydrostatic pressure in the tissue. In conjunction with a mechanically induced reduction in friction at the interface between the cell and the matrix, this pressure is capable of serving as a driving force for collective cell migration [146]. Thirdly, surface tension links forces to cellular functions, with the curvature forming a physical boundary that connects forces to cellular processes including growth, cell shape, and the creation of cell patterns [147]. How can the matrix surface tension affect interfacial tension? Firstly, the matrix surface tension functions as an elastic membrane. Surface tension causes the tissue surface to function like a stretched elastic membrane, thereby keeping the total surface area of the tissue to a minimum. Secondly, the matrix surface tension establishes pressure gradients. For example, a depression in the tissue surface caused from a meniscus enhances the pressure in that area. Thirdly, it encourages collective movement, as the interaction of cell–cell forces such as contractility and cell-matrix adhesion can be conceptualized as interfacial tension. This joint force is a key contributor to collective cell movement and the flow of tissue. This implies that both the curvature and surface tension of the matrix are crucial physical characteristics that determine the performance of a collection of cells. They cooperate to govern the global shape and dynamics of the tissue, and impact processes such as fusion of tissues, spreading, and their development.

### 4.1. Impact of Mechanical Forces on Mechanical Memory of Cancer Cells

Mechanical forces, which can be extrinsic and/or intrinsic, challenge primary solid cancers over a wide spectrum of biological sizes and various different timescales. These timescales span from fast molecular-level processes that are implicated in perception and transmission to more slowly occurring and large-scale processes, such as clonal selection, epigenetic modifications, cancer cell invasion, metastasis, and immunological defense [148]. Forces acting outside a cell originate in the ambient surrounding tissue and exert pressure on the cancer cell. Cells produce their own forces, which are referred to as intrinsic forces and are also known as autotonic forces, that affect the structures in their vicinity. These forces differ from the inherent mechanical characteristics of a cell or tissue, such as the stiffness of the ECM, as these characteristics influence how the tissue is deformed in reaction to a specific mechanical force [149,150,151,152,153,154]. In general, the mechanical characteristics of a cell or tissue are inherent and robust. Typically, it takes minutes, hours, days, or weeks for them to change, and they can be easily manipulated experimentally, for example, by altering the biochemical crosslinking of the ECM. While the fibronectin-crosslinking can elevate the stiffness of the ECM [10], the enzymatic breakdown of the ECM scaffold by matrix-metalloproteinases, such as MMP-14 or collagenase III can reduce the stiffness by local softening surrounding migrating cells [155]. Nevertheless, fibronectin crosslinking can also lead to an acceleration of stress relaxation irrespective of the decrease in stiffness [156]. In contrast, mechanical forces take effect over very short periods of time, sometimes only a few pico- or milliseconds [157] at the single bond level. Moreover, the binding and release of molecules or the folding of biomolecules also takes place within this time frame. The stress relaxation time of a collagen I matrix, such as within the tumor microenvironment (TME), varies from seconds to hundreds of seconds, based on the specific architecture of the matrix, involving fibril sliding (0.3 to 1 s), interfibril interactions (90 s), and the occurrence of a proteoglycan matrix (200 s), which represents the collective nature of a whole network [158]. In general, there is a tendency for relaxation time to rise with the degree of structural organization, from individual molecules (nanoseconds) to fibrils (100 s) to whole tissues (minutes). In addition, the forces to which a cell or tissue is locally exposed can quickly fluctuate in direction or intensity, for example in the course of the migration of cancer cells across a matrix scaffold [159,160]. Investigations into force reactions are frequently more challenging to conduct and evaluate [161,162,163]. At the tissue scale, this can be extremely intricate, since mechanical forces develop over time and spatial scale as a tissue expands or shrinks.

How does a variation in matrix relaxation time impact the spread of cancer? The degradability of collagen gel leads to significant alterations in stress relaxation following collagenase exposure. In addition, the relaxation time until half the maximum force is attained proves to be a potent predictor of cell spreading, emphasizing the importance of stress relaxation as a key mechanosensory cue in degradable matrices [156]. MMP activity instantly causes mechanical alterations in the ECM, which are then sensed by cells. The cells reacted to these alterations in relaxation by changing how they spread and their focal adhesions. In 3D systems, degradability also refers to the temporal progression of microenvironmental space generated by cellular MMPs, which facilitates alterations in cell shape, growth, and motility. As a result, cells in degradable scaffolds often show improved spreading in comparison to non-degradable scaffolds owing to the physical constraint provided by non-degradable matrices [164].

Mechanical stresses are categorized based on the deformation they induce and can include compressive, tensile, shear, bending, or torsional stresses. Compressive stresses have the tendency to compact the material, while tensile stresses have the tendency to elongate (stretch) the material. Shear stresses cause shearing and happen when a tangential force is exerted parallel to the surface of an object, such as cells or tissues. In contrary, bending and torsional stresses result in curvature or distortion of the cells or tissue. Mechanical stresses are relevant in biological systems and develop jointly, as local tensile stresses are counterbalanced by neighboring compressive stresses inside a cell [165] or in a tissue [166]. Continuous dynamic compressive stresses are part of natural physiology and normal healthy tissue growth [167,168,169,170].

Within the TME, both cancer cells and host cells are exhibiting abnormal physical tissue properties, among them modified tissue structure, elevated stiffness, increased IFP, and solid stresses [161] (Figure 4). Under the straightforward analogy of a spring following Hooke’s law σ = E·ε, tumor growth and pressure on the surrounding TME with elastic modulus $E^{\prime }$ lead to deformation $\varepsilon_{1}$ and a stress $\sigma_{1}$ [171]. As a result, the host tissue yields an equal and opposite stress $\sigma_{1}^{\prime }$, that is defined in terms of externally imposed solid stress ($\sigma_{1}$ = $\sigma_{1}^{\prime }$). This externally imposed stress, together with the growth-induced stress ($\sigma_{g}$), caused by mechanical reciprocal actions within the primary solid tumor, comprises the total solid stress transferred within the tumor [171].

The solid stresses are characterized by the mechanical forces transferred by the solid components of the tissue [161,162,172] and are completely different from the forces generated or transferred by fluids. Hydrostatic fluid pressure only shapes structures that expel water, like cell membranes (Figure 4). Cells are equipped with water channels that compensate for hydrostatic pressure over the membranes, so that the duration of the increased external pressure level results from the dynamics of the pressure increase and the relaxation rate achieved by the water channels. Nevertheless, relative to hydrostatic pressure, the implications of fluid flow have been more extensively investigated. Flowing fluids have been demonstrated to apply shear stress to the mechanosensors of endothelial and cancer cells, thereby influencing vascular viability and the metastasis of cancer [173,174]. In addition, solid stress, but not isotropic fluid pressure, can compact leaking blood and lymph vessels [175,176,177]. Cells moving within the crowded and frequently desmoplastic TME penetrate between cells by deforming themselves and through the structurally organized matrix [178,179]. In this process of restricted migration the cancer cells are also subjected to elevated compressive stresses, comparable to those imposed through increased solid-state stresses [180]. Nonetheless, these forces arise from movements caused by cells instead of external influences. There are, however, some biological events that are related to solid stress and restricted migration, such as epigenetic changes leading to reduced cell activity and dormancy or cell cycle arrest [181,182]. In addition, both confinement [183,184] and solid stress [161,172] have been widely reported in investigations where cells were entrapped in hydrogels that are either completely or partially non-degradable. In these types of models, cell movement is constrained because of the dense, porous matrix and cell compaction, preventing them from completely metabolizing or dissolving it [185]. When these cells multiply, they have to increase their volume, which causes them to press on the matrix, which results in the build-up of solid stresses [186].

### 4.2. Impact of the ECM Mechanics on Cancer Cell Mechanical Properties and Mechanical Memory

Similar to other cell types, cancer cells possess the capacity to adjust their (mechanical) properties to their mechanical environment, such as the stiffness, viscoelasticity and confinement of the nearby tissue like the ECM. Although stiffness has been selected as a mechanical characteristic in many cancer studies, it is not a good choice because stiffness is not constant in viscoelastic materials. Stiffness is time-dependent and impacted by strain-rate, materials history and temperature. For instance, the dissipation of energy due to structural alterations in a matrix is accountable for matrix softening. Conversely, the accumulation of residual stress in the matrix resulting from mutual interactions with cells leads to stiffening of the matrix. Both the migrating cell collectives and the ECM exhibit viscoelastic properties. The mechanical coupling of both systems and the interrelationship between their mechanical stores are interesting aspects to discuss. The mechanical coupling of migrating cell collectives and the viscoelastic ECM entails a dynamic, reciprocal transfer of force and deformation, in which cells restructure the matrix by exerting active forces like contractile forces (force transmission) that cause localized matrix restructuring. In turn, the matrix provides mechanical feedback. The alterations in the characteristics of the ECM, such as stiffness and topology, caused by cells function as mechanical signals that affect the cells and govern their direction of migration, velocity, and collective performance. There are also long-range effects. This coupled restructuring allows forces to be transmitted over long ranges across the matrix, thereby coordinating the behavior of cells that are far apart.

Due to their viscoelastic characteristics, i.e., their time-dependent mechanical response, which relies on the magnitude of the exerted force, both systems exhibit mechanical memory. Due to their elastic component, both the cells and the ECM react elastically when force is exerted and withstand sudden deformation. Hence, the cells and the ECM remember former forces by exhibiting elastic resistance to sudden alterations, but due to their viscous component, they tend to forget these forces over time because of viscous flow, which causes stress relaxation under constant strain and creep behavior under constant stress. This time-dependent response implies that they possess a memory of past mechanical stresses. The elastic component of the reaction mirrors recent forces, whereas the viscous component stands for history over longer periods of time. This interrelationship between their mechanical memories enables the transmission of long-range forces and the control of collective cell behavior via matrix restructuring. How are the mechanical memories of cells and the ECM connected? The interaction between the active mechanical memory of the cell collective and the passive viscoelastic memory of the ECM determines how forces are conveyed and how the whole system reacts to mechanical loads over time.

Is the matrix a stiffening/softening oscillator? The terminology “stiffening/softening oscillator” can be utilized to refer to a phenomenon in both cell mechanics and matrix mechanics. In cell-matrix interactions, it can characterize a process in which a cell-induced matrix initially softens and subsequently stiffens over time as a result of a cycle of matrix breakdown and reconstruction. In the early phases of interaction, there is an initial softening as cells can secrete enzymes that breakdown and thereby soften the ECM. Over time, progressive stiffening occurs, allowing the cells to reconstitute the matrix by deposition of increased collagen and other molecules. This can cause a significantly stiffer matrix, especially in the vicinity of the cells, as the fibers are more tightly packed and crosslinked. This whole cycle can be impacted through the mechanical tension on the matrix. A number of research findings indicate that synchronized mechanical oscillations between adjacent cells and the matrix may be a pivotal mechanism underlying how cells perceive their environment and uphold a tension set point [187]. In developing tissues, cells adjust the mechanical characteristics of their ECM in the presence of changing external loads to keep up a stress set point, but the mechanism involved remains unclear. It has been found that the set point represents a balance between cell-independent stress relaxation within the matrix and non-muscular myosin II-induced contraction of the cell. The matrix-dependent and cell-dependent phases display an oscillating tension component. Relaxation and renewed tension induce synchronization of mechanical oscillations in adjacent cells. It can be assumed that mechanical oscillation at the interface between the cell and the matrix is a core mechanism for the capacity of embryonic fibroblasts to perceive their mechanical surroundings, particularly the viscoelastic state of the relaxed tendon. This synchronized oscillating tension, engaging forces generated by the cells as well as external strain, assists the cells in sensing and reacting to tissue characteristics by forming a feedback circuit between the cells and the ECM [188]. The interaction of the pointed ends of extracellular collagen fibrils with the plasma membrane of fibroblasts to produce robust interfacial structures referred to as fibripositors occurs early in the development of mechanically stable tissue [188]. It can be assumed that a similar mechanism occurs in the TME with CAFs.

When cancer cells are subjected to a certain mechanical surrounding, they can alter their morphology, adhesion, and even gene expression to accommodate themselves more effectively. This adjustment can be maintained when the cells are transferred to a completely altered mechanical setting, which proves the existence of mechanical memory. An effective mechanism of mechanical memory is predicated on alterations in chromatin availability, which may be affected by mechanical forces and remain even when the forces are eliminated [189]. Activating and nuclear targeting of mechanosensitive proteins like Yes-associated protein/transcriptional co-activator with PDZ-binding motif (YAP/TAZ) [39], which can induce the expression of genes related to survival and migratory processes, provides another mechanism. Mechanical priming, in complementation to the retention of nuclear YAP, results in increased levels of microRNA-21, which remain after being grafted onto soft substrates, thereby keeping the mechanical memory [28]. It is supposed that mechanical memory has a major impact on cancer and its malignant progression. Hence, it can be postulated that mechanical memory is, in many ways, an important driving mechanism for the progression of cancer and may compromise cancer treatments. During cancer metastasis, cancer cells can “store and retrieve” the mechanical characteristics of the microenvironment of the primary tumor, for instance increased stiffness, enabling them to better withstand the stresses of metastasis and settle in remote organs (Table 1). In the case of resistance to medication, modifications in cell stiffness and other mechanical characteristics can influence the reciprocal reaction of cancer cells with medications and treatments, and possibly cause them to become resistant. Regarding the heterogeneity of cancer, mechanical memory may add to the multiplicity of cancer cells inside a tumor, increasing the difficulty of attacking all cancer cells in a single therapeutic approach.

Research findings indicate that mechanical memory is involved in the metastatic progression, whereby cells preserve adaptations that facilitate their survival and spreading to new sites. For instance, stiffness in breast cancer causes bone metastasis through the preservation of mechanical shaping [190]. Fibrotic matrix stiffness encourages pronounced metastatic phenotypes in cancer cells, which are retained after transfer to softer microenvironments like the bone marrow (Table 1). The sustenance of mechanical shaping is controlled by the Runt-related transcription factor 2 (RUNX2), which belongs to the family of osteogenic transcription factors and is characterized as a key player in bone metastasis. As a mitotic marker, it maintains the accessibility of chromatin at target sites.

### 4.3. Impact of ECM-Supported Mechanical Memory on Cancer Treatment Evolves over the Course of the Disease

**Figure 5:**
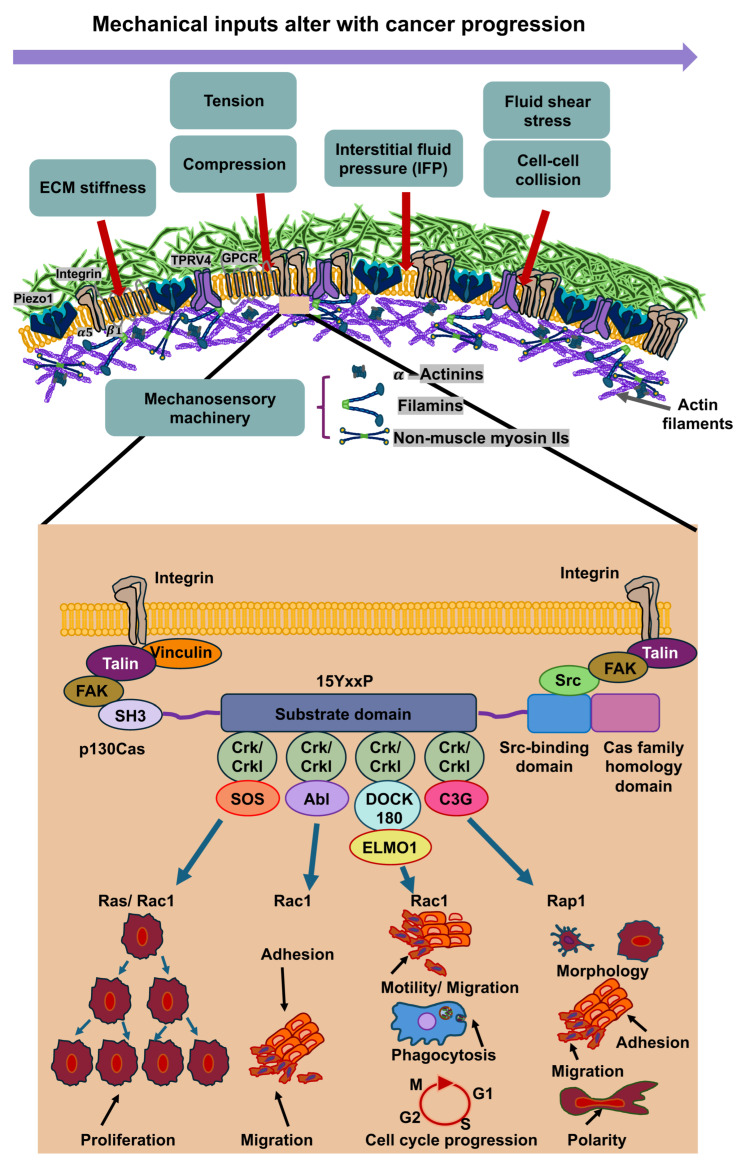
Mechanical inputs into cancer cells are s subject of change during the malignant progression of cancer. After mechanosensing of these mechanical cues through mechanoreceptors like integrins, ion channels (Piezo1 and TRPV4) and G protein-coupled receptors (GPCRs), such as GPR68. GPR68 (also synonymously referred to as OGR1) represents a G protein-coupled receptor (GPCR) that functions as a coincidence sensor for both extracellular acid concentration and mechanical stress, coordinating these cues to modulate cellular response, notably within the TME. When the mechanosensing process has been started via signaling through mechanoreceptors, the mechanosensory molecules underneath the plasma membrane of cancer cells, comprising α-actinin, vinculin, talin, filamins and non-muscle myosin IIs, are mechanically altered. The inset illustrates how p130Cas interacts with varies molecules to perform different functions.

There is no doubt that mechanical memory can have an impact on cancer treatment and therefore probably holds enormous therapeutic potential that is only just beginning to be explored. Therefore, a deeper comprehension of mechanical memory mechanisms could provide new therapeutic approaches to combat cancer cells in a targeted and efficient manner by interrupting or modifying these memory circuits or even avoiding the development of mechanical memory. As mechanical signals change during the malignant progression of cancer, their influence on mechanical memory can change. Consequently, the impact of mechanical memory seems to be dependent on the metastatic step and cancer progression (Figure 5). In Figure 5, the mechanical inputs on cancer cells can be altered during the progression of cancer, such as an increase in ECM stiffness, cellular tension, cell and tissue compression, elevation in IFP, rise in cell–cell collisions during cancer expansion and elevation in fluid shear stress due to compressed cancer vessels for circulating tumor cells (CTCs).

The alteration in mechanical inputs comes into play during the malignant progression of cancers, such as in cancer metastasis. Therefore, in the metastatic cascade, cancer cells experience various different mechanical cues over time and at the specific steps of the metastatic cascade that contribute finally to successful metastases formation as it is outlined below.

## 5. Potential Involvement of Mechanical Memory at the Different Steps of the Metastatic Cascade

**Figure 6:**
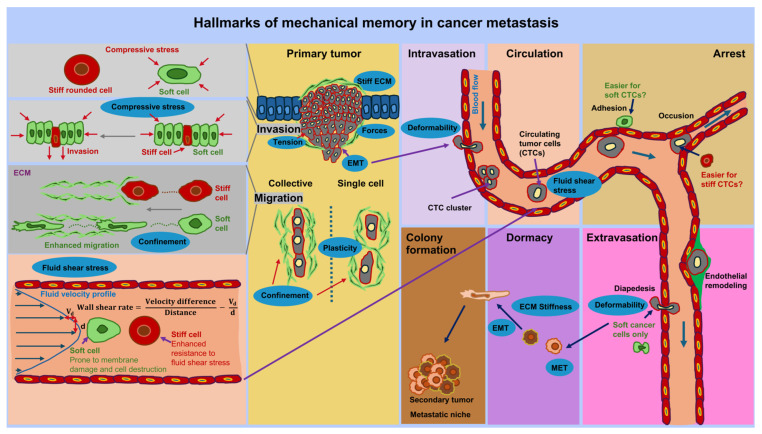
The effect of mechanical memory at each step of the metastasis cascade is illustrated. The metastatic cascade starts at the primary solid tumor at which cancer cells can either spread as individual cells or a collection of cells out of the tumor’s boundary into the tumor microenvironment (TME). The migrating and invading cancer cells reach vessels, such as blood vessels, in which they transmigrate (intravasation). In the vascular system, they become circulatory tumor cells (CTCs) that are exposed to mechanical stresses, such as fluid shear stress. Within the blood vessels CTCs can aggregate and adhere to the endothelial lining of the vessels. Endothelial cells can be remodeled due to external cues and possible through CTCs. There may also be individual dormant CTCs that remain in a dormant state, adhering to endothelial cells. Thereby, single or aggregated CTCs persist in a rather dormant state. At same time, some CTCs can transmigrate through the endothelium and the vessel walls into a targeted tissue site, which is referred to as extravasation step. At targeted tissue site these cancer cells can become dormant or migrate directly to their targeted site where they can transit form the mesenchymal to the epithelial state. These cancer cells proliferate and colonize the targeted site to form secondary tumors, which is referred to as metastasis. Mechanical memory can play a role throughout the entire metastasis cascade, for example in the form of stiffness, forces, and confinement. The hallmarks of mechanical memory are marked as blue ovals. Mechanical signals at the different steps of metastasis influence the characteristics of cancer cells, such as cell shape, and their behavior. Mechanical signals can become mechanical memory after prolonged exposure and hence foster the progression of cancer metastasis.

The metastatic cascade comprises successive steps that contribute to the spread of cancer, commencing with the transformative process of a primary cancer into an invasive tissue-invading phenotype. This ultimately results in malignant cells passing into blood or lymph vessels via transendothelial migration, a process known as intravasation of cancer cell [197]. The possible impact of mechanical memory is envisioned at each step of the metastatic cascade (see Figure 6). The metastasis cascade begins with the spread of specific cancer cells from the primary tumor, which can be induced by a changed mechanical environment such as stiffness and viscoelasticity. It is hypothesized that the migration from the primary solid tumor, intravasation, extravasation, dormancy, and metastatic colonization of cancer cells are influenced by the preceding mechanical encounters of cancer cells in the primary TME. This not only means that cancer cells perceive and respond to mechanical cues, but also that they preserve the biophysical adaptations imposed on them by these physical stresses after migrating to a new environment. In the following the impact of mechanical cues including mechanical memory is presented for the sequential steps of cancer metastasis.

### 5.1. Mechanical Conditions at the Primary Tumor

Solid malignant cancers display enhanced stiffness compared to healthy tissue or even benign tumors, such as those found in breast, pancreatic, liver, and prostate cancer [198,199,200,201] (see Table 2). It is noteworthy that the stiffness of cancers differs according to tissue type, depending on the stiffness of the normal surrounding tissue, varying between 1 kPa in the brain and 70 kPa in the bile ducts [161]. Compressive stress in kPa indicates how much force a material can withstand on a given surface area before it deforms or is damaged. A higher kPa value means that the material is stiffer and more resistant to pressure. A material with a compressive stress of 70 kPa is therefore significantly stiffer than one with 1 kPa. The conversion between the two units is: 1 mmHg = 0.133322368 kPa at 0 °C.

**Figure 7:**
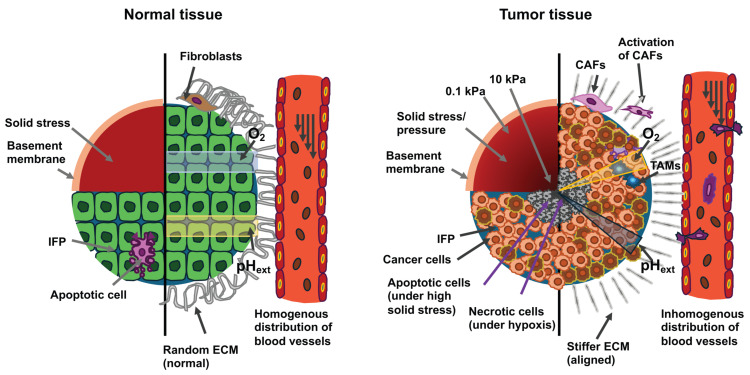
The drawings illustrate cross-sections of normal tissue (**left**) and cancerous tissue (**right**). Cells in cancerous tissue are subjected to high solid stress from structural elements and interstitial fluid pressure (IFP). The upper left quadrant of the two cross sections displays the distribution of solid stress. The lower left quadrant displays the distribution of IFP. Tumor tissue exhibits higher stiffness with a gradient that declines from the tumor center to the circumference and a higher IFP compared to normal tissue. High solid stress in the range of 4 to15 mmHg promotes the motility of cancer cells, while solid stress above 37 mmHg stimulates the apoptosis of cancer cells. The right half of both cross-sections depicts the constituent cells and the mechanical/chemical cues within the microenvironment. Compared to normal cells, cancer cells exhibit aberrant shapes and unorganized structures. The ECM inside a solid tumor is stiffer, denser, more interconnected, and more oriented vertically to the margin of the tumor in comparison to the loose, relaxed and isotropic ECM present in healthy tissue. Tumor-associated macrophages (TAMs) are also found within the tumor mass. The modified ECM promotes the development of tumors, invasion, migration, cancer metastasis, and the activation of cancer-associated fibroblasts (CAFs). Increased shear stress flow in the interstitial fluid of the primary tumor enhances the invasiveness and motility of cancer cells. Cancer cells can evade apoptosis, whereas normal cells are unable to escape it. The blood vessels in the primary tumor build a leaky and dendritic network that only penetrates the periphery of the primary tumor due to the strong limitation imposed by the center of the primary tumor. The hypoxia induced within the tumor by these abnormal blood vessels leads to an aberrant gradient of extracellular pH (pH_ext_) and necrosis of the cancer cells. In contrary, in normal tissue, blood vessels can perfuse the entire tissue and generate normal oxygen/pH_ext_ amounts.

The composition of solid primary tumors and their associated TME is well comprehended. The TME contains cancer cells and stromal elements, comprising ECM, basement membrane, vasculature, immune cells, and fibroblasts (Figure 7). During the course of cancer development and its advancement, all of its constituents undergo alterations in their physical structures and functionalities [221,222,223]. In many types of cancer, with only a few exceptional cases, primary tumors are generally more mechanically stiff in comparison to the healthy tissue from which they originated [161,208,221]. Mechanical brain cartography revealed that glioblastomas consist of both stiff and soft areas, which implies a high degree of heterogeneity within the tumor [208].

Stiffness levels were significantly decreased in glioblastomas, with a mean value of 1.32 ± 0.26 kPa in comparison to 1.54 ± 0.27 kPa in healthy tissue (*p* = 0.001) (Table 2). Nevertheless, some of the glioblastomas (5 out of 22) exhibited elevated stiffness [208]. Among the stiffer types of cancer are human breast tumors, which are five times stiffer compared to healthy tissue, with such increased stiffness positively associated with their malignancy [224]. The stiffness of human hepatic tissue is positively related to the incidence of hepatocellular carcinoma, with a threshold value of 20 kPa [225]. In addition to general stiffening, another striking mechanical feature of cancerous tissue involves the heterogeneity of its intratumoral stiffness [226]. Measurement using ultrasound elastography reveals significant spatial differences in tissue stiffness within breast and liver cancers [227]. In biopsies of human breast tumors, the TME is seven times stiffer (E = 5.51 ± 1.70 kPa) than the center of the tumor (E = 0.74 ± 0.26 kPa), while the stiffness of healthy breast tissue ranges from 1.13 to 1.83 kPa, which leads to the concept of the nanomechanical signature of solid cancers [203]. Besides stiffness, the viscoelasticity of cancerous tissue also deviates from that of normal healthy tissue. For instance, in vivo measurements using magnetic resonance elastography (MRE) demonstrate that in humans, the fluidity of benign meningioma tissue exceeds that of aggressive glioblastoma tissue by a factor of 3.6. This solid-like nature of glioblastomas eases their aggressive spread to the neighboring tissue [228].

Moreover, similar to other tissues, the TME possesses complex mechanical properties, such as viscoelasticity, which is the load relaxation over time [229], and mechanical plasticity, which means irreversible deformation due to stress [185], which have been reported to impact the propagation or migration of cancer cells. ECM restructuring through cancer cells or activated stromal cells can alter the mechanical characteristics of the TME [230,231,232]. Stromal cells frequently release transforming growth factor β (TGF-β), which leads to increased accumulation and crosslinking of ECM constituents including collagen, fibronectin, hyaluronic acid and laminin [233,234,235,236,237,238]. In combination, enhanced ECM depositing and crosslinking increases the stiffness of the ECM, which impacts the phenotypes of the cancer cells [161]. Elevated stiffness stimulates the activation of fibroblasts and encourages myofibroblast phenotypes that are positive for alpha-smooth muscle actin (α-SMA). α-SMA is encoded by Acta2 which is frequently coupled with the myofibroblast phenotype, that is triggered through the fibronectin isoform extradomain A fibronectin (EDA-FN) [239]. In contrary, in several fibrotic diseases, Acta2 and other contractile hallmarks are reduced, whereas EDA-FN secretion is elevated. Thus, the relationship is intricate and contextual, such as EDA-FN may promote the transition toward a contractile cellular phenotype. This creates a positive feedback circuit in which elevated levels of myofibroblast activation promote ECM secretion, ECM crosslinking, and contractility of myofibroblasts, leading to continued stiffening [240]. Myofibroblast-like cancer-associated fibroblasts (CAFs) are commonly regarded as cancer-promoting [241,242], however, some evidence suggests that they may also have anti-tumor effects according to fibroblast type and tissue context [243,244]. This is illustrated by a study that revealed hyaluronan secreted by CAFs has a tumor-promoting effect, whereas collagen I secreted by CAFs has a tumor-inhibiting effect [245]. The components and stiffness of the ECM may also have an implication for immune cells [246]. For instance, a collagen-rich, rigid ECM interferes efficiently with the infiltration of T-cells [247] and decrease the cytotoxic activity of them [248]. Nevertheless, it is uncertain whether increased ECM stiffness stimulates the polarization of M1 macrophages, which are pro-inflammatory and tumor-suppressing, or M2 macrophages, which are tumor-promoting [249,250]. Moreover, matrix stiffening decreases vascular proliferation and vascular integrity [251], and changes in the mechanosensory properties of endothelial cells via the Rho signaling route may account for vascular malfunction in the TME [15,252]. The stiffer microenvironment encircling primary solid cancers lead to the development of specific cancer cell phenotypes that can disseminate out of the primary cancer into the surrounding tissue. When cancer cells are exposed to increased stiffness in their primary cancer’s microenvironment beyond a certain threshold and/or for a certain duration, they can store this as mechanical memory. This acquired mechanical memory could help cancer cells to migrate efficiently to their target tissue for metastasis. Whether it also plays a role in selecting the target tissue for metastasis is still unclear, but it is quite possible. Moreover, this mechanical memory, a sustained adaptation of their cytoskeletal dynamics, improves their capacity to infiltrate and survive in new surroundings.

### 5.2. Cancer Cell Migration Through the ECM Microenvironment

The association between substrate stiffness and cancer cell migratory capacity is linked to the metastatic capacity of cancer cells, which is characterized by a mesenchymal or epithelial phenotype (Figure 4) [253]. Recently, an intriguing classification of cancer cells by Wang and Yan [254], which is presented in the following. Metastases are generally known to be accompanied by epithelial-mesenchymal transition (EMT), which enables epithelial cancer cells to adopt mesenchymal characteristics and thereby achieve migratory capabilities [255,256]. Mesenchymal-epithelial transition (MET) refers to the change in cancer cells from a mesenchymal-type to an epithelial-type form and restore proliferative capacity. Epithelial-mesenchymal plasticity (EMP) permits cancer cells to switch between several states of the epithelial-mesenchymal spectrum [257], which promotes the migration of cancer cells and their consecutive colonization of metastastic niches. The hysteresis of EMT concerns the retention of the mesenchymal state in mesenchymal cancer cells even after leaving the microenvironment that stimulates EMT [258]. The term hysteresis therefore denotes the phenomenon whereby the state of a system relies on its evolution/history, suggesting a type of memory process, similar to cellular memory, which may also be based on a mechanical memory in terms of increased stiffness. Hysteresis is linked to aggressive cancer and a worse prognosis [258]. The hybrid epithelial/mesenchymal phenotype is defined by cancer cells exhibiting both epithelial and mesenchymal characteristics. Cancer cells in this hybrid state tend to be extremely aggressive and are linked to the cancer stemness phenomenon [259]. On the basis of EMP, EMT hysteresis, and the hybrid epithelial/mesenchymal phenomena, a classification for cancer cells with EMP capability has been propounded [254]. This classification is intended to evaluate the performance of cancer cells throughout the spreading stage. This classification categorizes cancer cells with EMP potential into four groups, each with distinct biological properties and propagation characteristics, leading to different prognoses for the cancer. The first group comprises irreversible hysteresis-type cancer cells that can induce stochastic metastasis and display an oligometastatic and metachronous type of metastasis. The second group comprises weak hysteresis-type cancer cells that can give rise to distant metastases that are less effective. The third group covers highly hysteresis-type cancer cells that can take on a temporary hybrid epithelial/mesenchymal phenotype and are able to undergo effective metastasis. The fourth group encompasses stable cancer cells exhibiting a hybrid epithelial/mesenchymal phenotype, which tend to be highly aggressive and capable of metastasizing fast and widespread.

Apart from biochemical cues, EMT can be induced by mechanical cues. For example, a highly invasive oral squamous cell carcinoma cell line (SCC25) traveled more rapidly on stiffer substrates (20 kPa versus 0.48 kPa) on a collective and individual migration basis, while a less invasive cell line (Cal27) showed no sensitivity to substrate stiffness [253]. Extended contact with stiff substrates caused less invasive cancer cells to switch to EMT and subsequently develop stiffness-reliant movement [253]. In fact, matrix stiffness and the consequential forces exerted on the cell nucleus encourage the translocation of transcription factors participating in the EMT process, which are pivotal in the spread of cancer cells [260,261,262,263]. After dissemination from the solid primary cancer, the migration of cancer cells is confined within the TME, such as local geometry (confinement). It has been shown that the restriction can cause a switch between two cell types, known as EMT or partial/hybrid EMT, thereby encouraging the penetration of cells between long and tight gaps [101,264].

### 5.3. Intravasation

When cancer cells penetrate the vascular system, they have to resist the circulatory forces of the blood and escape surveillance by the immune system (Figure 4). After the transmigration event, in which cancer cells squeeze usually as single cancer cells through the intercellular junctions of endothelial cells, such as their tight junctions, cancer cells are largely deformed and usually polarized [265]. During the transmigration step, the cell nucleus is deformed and squeezed as well. All of which contributes to a mechanical adaption and subsequently may lead to mechanical memory in cancer cells that may keep them primed to for other mechanical adaption, such as in the circulation, cancer cell extravasation or colonization of the metastatic niche.

### 5.4. Circulation and Arrest

Circulating tumor cells (CTCs) subsequently attach to or undergo physical entrapment within a distant microvascular lumen (Figure 4). These CTCs can be described by a mechanical conditioning (MeCo) score that represents a multigene expression signature. Cancer cells acquire MeCo in the primary breast tumor, mirroring their reactivity to ECM stiffness imposed through tumor fibrosis. Chromatin reorganization following mechanotransduction enables cancer cells to preserve these learned aggressive features even without mechanical pacing from the primary TME, for example, after spreading through the circulatory system while undergoing the metastatic process. It is important to note that patients with tumors with a high MeCo score have a higher chance of suffering from metastatic breast cancer than patients who have a low MeCo score. In addition, CTCs are linked to a faster rate of metastatic spread, which renders the detection of CTCs in the bloodstream of breast cancer patients an important prognostic indicator for metastatic progression in breast cancer. Apart from their count per unit of blood volume, the specific prognostic characteristics of CTCs have not yet been thoroughly researched. Patient-specific biopsies will be used to determine whether MeCo levels rise gradually alongside the metastasis cascade, from the primary cancers to CTCs all the way to distant metastasis. It has been demonstrated that the MeCo score tends to be higher in CTCs compared to primary tumors and higher in METs when compared to CTCs, in both early and late stages of breast cancer, that it can be said that primary tumors possess a lower MeCo score compared to CTC that exhibit a lower MeCo score than METs [266]. Therefore, the MeCo score increases throughout the entire metastasis pathway in breast cancer. These results reveal that mechanical conditioning by primary solid tumors is preserved during metastasis after mechanical stimulation by the stiffness of the ECM is dissipated when the cancer cells spread via the circulatory network. The mechanical conditioning is a specific kind of mechanical memory. These findings also provide evidence for the hypothesis that cancer cells with higher MeCo levels have a greater ability to metastasize and may be recruited for metastasis. It is important to note that these results yield a new characteristic of CTCs, namely the MeCo value, which is an indicator of increased metastatic potential. Since CTC-MeCo levels are elevated even in the early stages of breast cancer, they could serve as a powerful prognostic marker alongside CTC numbers to advance the evaluation of metastasis potential in the early stages of cancer.

### 5.5. Extravasation

After entering the vascular system, cancer cells translocate from the vascular compartment into the peripheral tissue through transendothelial migration, which is referred to as extravasation of cancer cells (Figure 4). Several of the biophysical adaptations gained in the primary tumor are anticipated to be preserved through the mechanical memory of cancer cells and may augment the extravasation of CTCs into the secondary target site. Cancer cells produce elevated intracellular pressure by contracting the posterior cortex to ease nuclear trafficking through narrow paths like matrix pores and the intercellular space, such as tight junctions between endothelial cells [267,268]. They also necessitate the involvement of integrin β1 when they extend protrusions between endothelial cells to attach to the basement membrane and subsequently extravasate [269]. Elevated ECM stiffness induces integrin β1 signaling [59,204,270,271,272], an effect that has been previously demonstrated in in vitro models of mechanical memory. Therefore, it is hypothesized that the maintenance of activated integrin β1 through mechanical memory, albeit solely partial, probably augments extravasation. Whereas the retention of integrin β1 activation has not been directly characterized in mechanical memory experiments, MCF10A cells migrating on soft, e.g., a 0.5 kPa value, polyacrylamide hydrogels, sustained elevated focal adhesion area, actin alignment, and MLC phosphorylation two days after being placed on stiff, e.g., a 50 kPa value, hydrogels [191]. There is also an indirect connection with mechanical memory, as the extravasation mechanism is further enhanced through reshaping of the vascular network by the release of angiogenic factors and MMPs by cancer cells [273,274,275]. Notably, breast cancer cells grown on stiffer 2D silicone substrates, such as 1.1 MPa and 14.3 kPa versus 5.2 kPa, demonstrated higher transendothelial migration in a soft 3D hydrogel in vitro, which was concomitant with enhanced MMP9 release, implying that the increased migration in reaction to stiffness was maintained and perhaps facilitated through MMP9 [276]. In addition, cancer cells enhance VEGF synthesis in reaction to the stiffness of the primary tumor, as demonstrated in rat models of liver cancer and human liver cancer cell lines grown on stiff 2D hydrogels [277]. This may also raise the penetrability of the vascular interface, thereby intensifying the extravasation.

It has been revealed that stiffness-imposed increased migration is maintained via a mechanical memory that includes the actomyosin and focal adhesion regulation in the MCF10A, MCF7, and A431 cell lines propagated on 2D polyacrylamide hydrogels [191]. Since extravasation by definition represents a transendothelial migration, maintaining an increased migration phenotype presumably favors extravasation. In addition, force production, a prerequisite for migration, is particularly crucial for the extravasation of cancer cells [17,278,279]. It has been shown that inhibition of MLC kinase (MLCK) and small GTPases, one of which is ROCK, in breast tumor cells impaired force production, which diminishes transendothelial migration [16]. In a trial on mechanical memory, fibroblasts grown on 100 kPa substrates and then transferred to 5 kPa substrates demonstrated improved contractility in comparison to fibroblasts grown entirely on 5 kPa substrates [280]. In addition, increased MLC phosphorylation was maintained in MCF10A cells that were transferred from 50 kPa to 0.5 kPa hydrogels [191]. Enhanced cell contractility can therefore be sustained and is expected to amplify the extravasation process. In addition, maintaining ideal level of cancer cell stiffness would enable the cancer cell to concurrently withstand the high fluid shear stresses inside the vasculature and squeeze through the endothelial cell–cell junctions avoiding lethal nuclear injury. The perpetuation of cell stiffness subsequent to priming on stiff substrates has not yet been demonstrated. It seems relevant to investigate this in cancer cells to gain insight into whether the mechanical characteristics of the cells due to stiffness can impact the metastasis to distant targeted tissue locations. Therefore, it can be hypothesized that while the stiffness of cancer cells generally adapts to the stiffness of the new tissue microenvironment, the stiffness of the cells can also be partially retained, which would support the mechanical memory effect.

### 5.6. Dormancy

When these individual cancer cells finally reach the targeted organ, they are unable to switch to the mesenchymal mode and are not capable of proliferating (Figure 4). Consequently, these individual cancer cells stay dormant within the target organs lacking proliferation and are incapable of initiating colonization, thus turning into sleeping tumors, which are referred to as dormant tumors. Random noise, fluctuations within cancer cells, or MET-stimulating cues from the TME can emerge after a specific period of time and trigger the MET process. These arrested cancer cells can convert to an epithelial state, reacquire their proliferative capacity, induce metastatic colonization, and give rise to distant metastases. Since MET is a stochastic process, its efficacy is low (fewer metastatic lesions) and it takes time to appear (metachronous), resulting in an oligometastatic and metachronous metastasis profile. According to epithelial-mesenchymal plasticity, notably the hysteresis of epithelial-mesenchymal transition and the hybrid epithelial/mesenchymal phenotype, a classification of cancer cells has been suggested, which indicates that cancer cells with epithelial-mesenchymal plasticity can be categorized into four distinct types [254]. Type-1 cancer cells exhibit irreversible hysteresis and can stochastically generate metastases, displaying an oligometastatic and metachronous metastatic profile. Type-2 cancer cells are characterized by weak hysteresis and can give rise to low-efficiency distal metastases. Type-3 cancer cells exhibit intense hysteresis, temporarily adopt a hybrid epithelial/mesenchymal phenotype, and metastasize in an effective manner. Type-4 cancer cells have a stable hybrid epithelial/mesenchymal phenotype, are highly aggressive, and are capable of performing fast and extensive metastasis [254]. These four types of cancer cells had varied biology, spreading features, and prognoses. Type-1 cancer cells are considered less aggressive compared to the other types because of their weak EMP. Consequently, it was proposed that oligometastatic and metachronous metastases offer a better chance of survival than non-oligometastatic and synchronous metastases, which may be attributable to the presence of Type-1 cancer cells in metachronous metastases and oligometastases, which exhibit low EMP and a less aggressive capacity [254]. Importantly, biophysical adaptations of cancer cells learned in the primary tumor and maintained through mechanical memory may also govern cell quiescence at secondary locations. Cancer cells that enter a distant organ are inactivated because they are unable to bind integrin-β1 and trigger the activation of FAK [269,281], and because they encounter unfavorable biochemical and mechanical signals when they reach the secondary target site. Notably, it has been found that higher matrix stiffness, such as about 1 kPa versus 0.09 kPa, in various cancer cell types in 3D fibrin hydrogels triggered dormancy, which was sustained through an epigenetic pathway involving downregulation of integrin β3 [282]. These stiffness values are too low to replicate the stiffness of cancerous tissue and were selected as they correspond to the stiffness of cells. Nonetheless, even a slight elevation in hydrogel stiffness was capable of trigger dormancy. While these findings indicate that dormancy can be perpetuated epigenetically, stiffness was regulated by enhancing fibrinogen levels in the hydrogels and consequently by enhancing the density of adhesion ligands at the same time, suggesting that dormancy cannot be solely accounted for by mechanical signals. In fact, the results could not be reproduced with collagen I using the identical cells in the same model [282]. A mouse model of pulmonary fibrosis has revealed that elevated collagen I accumulation in the lungs induces the proliferation of quiescent mouse breast cancer cells (D2.0R) that are injected into the tail vein through integrin-β1 signaling paths and the subsequent activation of the proto-oncogene tyrosine protein kinase Src, FAK, ERK, and MLCK [283]. These signaling routes are frequently activated in reaction to collagen accumulation and increased matrix stiffness within the primary TME [59,204,270,271,272]. Consequently, the preservation of these mechanisms through mechanical memory could preclude cancer cells from entering a dormant mode when they reach the target location for metastasis. To verify this, the following experiment can be proposed in which non-quiescent cells that are mechanically preconditioned on soft or stiff substrates need to be injected into mice and their proliferation (or lack thereof) observed once they reach a metastatic site that tends to stimulate quiescence, such as the bone marrow. This experiment is intended to provide evidence that the mechanical training to which cells are subjected on a specific substrate can impact their performance, which involves their ability to multiply or transition into a dormant state, even when they encounter a new environment that would naturally inhibit their multiplication. However, for many approaches to combating cancer that are based on preventing cancer cells from invading metastatic sites, this would be even worse. Specifically, it would not be possible to relocate dormant breast cancer cells back into the circulatory system, while their return to the tissue could be prevented (by inhibiting CXCR4 and E-selectin), thereby progressively eradicating micrometastases from the bones in a minimally toxic way [284]. A novel approach to targeting dormant micrometastases in the peripheral circulation could therefore be used not only to eliminate and/or trap inherently resistant breast cancer cells, but also to enhance the efficacy of present adjuvant therapies. Without the knowledge of the impact of mechanical memory, this approach may not be fully successful. Finally, Price and colleagues have closed a knowledge gap by postulating, first, the difference in the time frame between the response of cells to mechanical signals and the development of mechanical memory and, second, the difference in persistence time between the duration of the cell’s initial response to mechanical stimuli and the persistence time of the developed mechanical memory through dynamic self-reinforcement of gene expression in cells [25]. In the future it needs to be explored how the impairment of mechanical memory can support these cancer treatment outcomes and finally lead to a benefit for the patient.

### 5.7. Colony Formation

Recently, it has been hypothesized that biophysical adaptations that are mechanically triggered in the primary TME and maintained via mechanical memory can also modify colonization of the remote targeted organ [285]. It is critical that this theory is consistent with the three requirements for a successful metastatic colonization. The first requirement is the restoration of tumor growth through the self-renewal of cancer stem cells. The second requirement is the adjustment and installation of organ-specific colonization schemes. The third requirement is the constitution of a microenvironment that promotes metastatic colonization [286]. Specifically, cancer cells improve their surveillance [217,270,287,288], stem cell-like properties [287], and multiplication [270,289,290] reaction to increased matrix stiffness within the primary TME. After all, cancer cells spread to a remote targeted tissue and multiply there to develop secondary tumors, which is known as colonization. The ability to populate a secondary site underscores the adaptability of cancer cells to different physical stresses, such as a stiffened ECM and basement membrane, tight junctions, and fluid shear stress, each of which modify the mechanical phenotype of the metastatic cancer cells. When maintained at the secondary location, enhanced cancer stemness may endow cancer cells with the capacity to reactivate tumor growth, which is the primary requirement. In addition, maintaining an improved survival cell characteristic could assist cancer cells in surviving the harsh microenvironment of the secondary site. There is a requirement for experiments to determine whether the survival and stem cell characteristics induced by stiffness can be sustained from the primary TME to the secondary target site. Adjustment to the particular microenvironment and cell communities within the secondary organ, as well as the installation of organ-specific colonization schemes, is the second requirement and may also be underpinned by the persistence of mechanically imposed phenotypes. Most notably, it has been demonstrated that stiffness-induced release of osteolytic factors stimulates bone colonization patterns in vitro [291] and in vivo settings [190]. A breast cancer cell line grown in stiffer 3D alginate-gelatin hydrogels, such as 6 to 10 kPa instead of about 2 kPa, and subsequently dissociated exhibited enhanced secretion of osteolytic factors when grown in 3D-printed bone-like scaffolds [291]. In addition, stiffness-facilitated activation of RUNX2 in breast cancer cell lines was preserved following cell injection into mice and enhanced osteolytic bone reseeding and metastasis [190]. Moreover, substrate stiffness can promote the release of soluble factors like MMP-7 and MMP-9 [249,271,277,292], and the release of growth factors, cytokines such as CXCL12 and matrix-remodeling enzymes, has been proven to coordinate stromal cells such as fibroblasts at the secondary site to aid in metastatic site colonization [293,294]. It is probable that mechanically stimulated release of several soluble substances could encourage metastatic site colonization, which is the third prerequisite. More research is required to clarify whether, and with what degree of intensity and over what period of time, the biophysical adaptations obtained in the primary TME remain in place at the distant location. If these hypotheses are confirmed by experiments, the mechanical memory of cancer cells could be a key mechanism for effective cancer metastasis. Therefore, mechanosensory systems, mechanotransduction processes, and mechanical reciprocity are key players in all phases of the metastatic cascade, encompassing the emergence, progression, and spread of cancer. This raises the question of whether mechanical influences affect genetic and epigenetic expression to such an extent that they actually alter the response to drugs. This point will be addressed in the following section.

### 5.8. Mechanical Input Affects Drug Responses via Genetic and Epigenetic Expression

Mechanical effects have such a strong impact on genetic and epigenetic expression that they can modify the response to drugs, particularly in certain tissues. As mentioned earlier, this field of research is referred to as mechanotransduction, which is the process whereby cells translate mechanical signals into biochemical reactions. These cellular reactions can considerably influence the metabolization of a drug or how tissue reacts to an active pharmaceutical substance. Firstly, cellular reactions include alterations in gene expression. For instance, mechanical forces such as shear stress, compression, and tension can directly induce signaling paths that modify the activity of transcription factors, these in turn controls the gene expression. Moreover, the translocation of transcription factors into the nucleus can be induced. Inside the nucleus they intact with the DNA and thereby control expression of genes. This can result in varying concentrations of proteins implicated in the absorption, delivery, metabolism, and clearance of drugs. Secondly, epigenetic modifications occur, because mechanical cues can induce epigenetic alterations, such as by altering chromatin accessibility. For instance, it has been reported that mechanical signals can cause alteration in histone, which are referred to as histone modifications. A change in the winding density of DNA around histone proteins is able to increase or decrease the accessibility of specific genes for transcription. Another example is that the methylation of DNA can be modified. Thereby, the methylation patterns on DNA may change, which can result either in repression or activation of gene expression. Thirdly, there are impacts on the metabolism of drugs as the regulation of genes encoding drug-metabolizing enzymes and transporters can be strongly altered via mechanotransduction. For instance, in a spöif primary tumor, mechanical stress induced through high interstitial fluid pressure and a stiff ECM can modify the expression of drug efflux carriers. This results in decreased absorption of drugs by cancer cells and is an essential mechanism of chemoresistance. Fourthly, the effects of mechanical influences are highly context-dependent and manifest themselves in unique ways in different tissues. Abnormal mechanical characteristics in a tumor can trigger epigenetic alterations that drive disease advancement and enhance resistance to drugs. In healthy tissue, mechanotransduction is crucial for physiological processes like bone reorganization and the integrity of blood vasculature.

What are the direct effects of drug resistance? The stiff mechanical microenvironment of multiple primary tumors has been shown to act as a factor promoting resistance to medication [295]. In fibrotic diseases, including cancer, tissue stiffening caused by the build-up of collagen can impact how drugs are transported and processed. Modified blood flow and vascular stiffness can impact gene expression within the endothelial cells lining blood vessels, thereby influencing the efficacy of medications. Mechanical forces can affect the expression of genes that are critical for drug metabolism and transportation, comprising cytochrome P450 enzymes and ABC transporters. Modifications to these genes can result in differences in the absorption, distribution, metabolism, and excretion of a drug, which may change its efficiency and the likelihood of side effects.

A prerequisite for devising novel and effective methods for the diagnosis, prevention, and therapy of cancer is therefore a profound comprehension of metastasis, encompassing not merely the genetic, molecular, and biochemical underpinnings, but also the key mechanical features that go beyond recognition of mechanical signatures [9] and aim to identify the role of mechanical memory.

## 6. Molecules Are Involved in Dynamic Regulation of Mechanical Memory (MeshCODE Theory)

Mechanical memory can occur at various levels, for example at the level of focal adhesion/scaffolding proteins such as talin and vinculin. Cells can use a computational circuit referred to as MeshCODE, which is a network of binary switches in talin (13 switches) [296]. The MeshCODE theory hypothesizes that the scaffold network of talin and vinculin molecules at the synaptic junctions constitutes a sophisticated mechanical processing and storage (memory) apparatus [297]. Talin incorporates a set of force-dependent binary switching mechanisms, and each domain switching condition results in quantized step alterations in talin length in the micrometer range. These talin domains can be reversibly switched between two thermodynamically stable conditions, such as a folded and an unfolded state, when mechanical force is applied [297]. A key finding that contributed to the MeshCODE theory is that the switching events in talin possess mechanical hysteresis [99] which implies that both the folded and unfolded condition of each domain represents a thermodynamically stable state provided that the talin undergoes a mechanical coupling between the integrin-ECM complex and the actin cytoskeleton [297]. This endows talin with a novel feature in molecular memory, as each talin molecule can be engraved with a permanent pattern of switching conditions which are the specific outcome of the forces exerted on it [297]. The conformational state of each switch dictates what type of signaling molecules are enlisted, thus instructing the cell differently depending on the force applied, allowing talin to act as a mechanosensitive signaling mediator, which is referred to as mechanosensitive signaling hub [83].

In the past, the average diameter of a dendritic spine is 1 μm, so this analysis identifies a valid gear-like mechanism for the dynamic control of synaptic functioning, where the placement of enzymes and substrates in relation to each other is mechanically codified through the MeshCODE switching schemes, which could govern synaptic transmission, such as in the brain. The question is whether the MeshCODE could also serve as storge mechanism for mechanical memory in a similar manner as it seems to be for synaptic functioning in the brain. In contrast to talin, vinculin itself seems to harbor no set of force-dependent binary switches but is enlisted through force-dependent conformational alterations in other proteins such as talin and α-catenin. In addition, talin-activated full-length vinculin interferes with actin [298] which generates tension, triggering conformational changes in vinculin and its further activation [299]. Vinculin, that ties to nine of the unfolded talin switches, can act to deliver further stabilization to the unfolded condition [300,301], thereby constraining refolding and reinforcing these linkages. Therefore, vinculin is assumed to function as a molecular coupling (clutch) that connects the actin meshwork to emerging nascent adhesions and focal adhesions [302,303,304,305]. Vinculin can attach to several proteins in cell structures that are linked to adhesions or the cytoskeleton. VBS of proteins such as talin, α-actinin, or α-catenin are not exposed unless their actin-binding domain (ABD) is bound to the F-actin cytoskeleton. Engagement with F-actin and force-induced unfolding of the helical domains uncover the VBS. Vinculin engagement with VBS activates it by causing its head to detach from the tail domain. The free vinculin tail is then able to either interact with the F-actin cytoskeleton or attach to PIP2-enriched membranes. This means that a large number of relative pulling directions between the strain gauge proteins and vinculin are conceivable. Moreover, vinculin remodels the organization and dynamics of the Arp2/3 complex-facilitated branched actin reticulation. In specific detail, it has been revealed that vinculin can bundle dendritic actin reticulations through fast tethering and crosslinking of actin filaments. Cryo-electron tomography has revealed that vinculin enables stable yet flexible actin bundles that display mixed polarity organization [306]. Various VBSs from various non-homologous actin-binding proteins consist of conserved helix motifs that connect to the vinculin head domain [306]. They thus utilize the identical mechanism to govern directed force transmission. Vinculin therefore acts as a signaling hub with a large number of binding molecules and is especially famous for its capacity to physically strengthen the connections between actin filaments and adhesion proteins like talin, α-actinin, or α-catenin [307]. These proteins include VBSs that are concealed in mechanically unstable helix bundles and are uncovered during force-facilitated unfolding. All of this appears to provide a mechanism whereby mechanical memory can be retained in an efficient manner [301,308,309,310]. Vinculin’s partner proteins feature folded helical bundle domains that function as binary switches and undergo unfolding when mechanical tension is applied, thereby uncovering new attachment sites. Vinculin then attaches to these uncovered areas, thereby encouraging the formation and stabilization of cell adhesions. In addition, vinculin is controlled by force, such as when tension is applied to the cytoskeleton of cells that generates a pulling force on the focal adhesions. The force is also able to unfold domains within talin, uncovering vinculin binding sites that were formerly concealed inside the folded protein. Vinculin attaches to these uncovered sites, altering the conformation of the protein and encouraging more powerful interactions inside the adhesion complex. Different types of binding partners can engage with the protein in its folded (inactive) or unfolded (active) form, thereby creating a binary switch that is controlled by force.

Binary switches present in α-catenin relate to its mutually exclusionary engagement with the cadherin-β-catenin complex and F-actin [311]. E-cadherin and α-catenin serve as tumor suppressor proteins [312,313,314]. The deregulation of the transcription coactivator YAP provides a mechanism by which the removal of the cadherin-catenin complex can impact on growth of tissues. The Hippo signaling cascade was identified in Drosophila melanogaster and turned out to be a key regulatory pathway in tumorigenesis. This signaling pathway consists of STE20-like kinases MST1/2, Salvador homolog SAV1, MOB kinase activator 1 A/B (MOBKL1A/B), Yes-associated protein (YAP), and WW domain-containing transcription regulator 1 (TAZ), as well as members of the transcription enhancer of activators domain (TEAD) family [315]. The Hippo signaling pathway not exclusively regulates tumors, but also has a key role in cell growth, proliferation, differentiation, embryonic development, regeneration of tissues, homeostasis of organs, and injury repair. Latest investigations have demonstrated that upstream signal control is highly intricate and encompasses cell polarity, mechanical stress, cell density, soluble agents, and external stress [316]. YAP and TAZ stimulate the growth of tumors, whereas MST1/2 and LATS1/2 impede tumor development. Disturbance of the Hippo signaling route is linked to cancer development, metastasis, and resistance to medications, and represents a possible candidate for cancer treatment [317]. YAP is the key effector of the Hippo pathway that is known to be dysregulated in multiple cancer types, such as breast [318]. In healthy tissue, the Hippo pathway, in which Hippo is activated, keeps a state of homeostasis as it serves as a regulator for cell growth and organ size, whereas in cancerous tissue Hippo is inactivated and hence promotes the progression of tumors [319]. Yap is affected in cancer cells of the primary solid tumor by the general compression of tumors and the stiffening of the tumor environment.

Tissue stiffens when compressed. This property can intensify in the event of tissue injury or cancer, which this property is used during palpation of tissues as part of a diagnosis, for example when searching for nodes during a cancer screening. However, this stiffening reaction has been a long-standing biomedical paradox. Tissue is made up of cells contained within an intricate mesh of fibers, and compressing the ends of a cord should decrease tension rather than increase it. Once tissue is compressed, the cells in the interior elongate laterally, tugging on the fibers connected to them and exerting increased total tension on the tissue architecture [320]. Targeted manipulation of the proteins that attach cells to the ambient fiber network could thus be the ideal approach to decreasing the overall stiffness of the tissue, which is a key objective in the therapeutic treatment of cancer. In contrast, the fiber networks inside and encircling the cells react in the opposite manner to the tissue. A search for the basis of this relationship began with experiments on reconstituted fibrin networks that are free of cells. Unlike natural tissue, these cell-free fibrin scaffolds, exhibit no stiffening when compressed. This suggests that the main reason for tissue stiffening appears to lie in the cells, which try to maintain their volume. A cell can be crushed and deformed, but it always occupies the identical amount of space. However, these reconstituted networks contain solely water in place of cells. They exhibit a behavior that is similar to a sponge: when compressed, the water simply escapes. When Polymer networks are compressed, they become softer. In contrast, when they are stretched, they become stiffer. When polymer networks are compressed, they become softer. Moreover, these materials can be transformed into ones that become stiffer when compressed but not when stretched by incorporating either cells or inert particles into the network to limit the relaxation modes of the surrounding fiber networks. Specifically, inclusions of particles inhibit the stiffening effect when subjected to shear stress. When the volume fraction of particles is relatively low, they influence the elasticity of the polymer networks to a minimal extent. However, when the particles are packed more densely, the material shifts from compressive softening to compressive stiffening [320].

The fibers themselves become stiffer under tension and softer under compression. This behavior is quite understandable, because when a wire is stretched, it becomes taut. However, if you press down on a rubber ball, it becomes shallower and the equator becomes wider. The individual cells react to pressure on the tissue by stretching the fibers attached to them sideways. Another traditional paradox is that although most tumors like breast and colon tumors, are stiffer compared to healthy tissue [36], individual cancer cells are generally softer compared to healthy cells [321]. Since cancer cells are softer, they are more likely to deform under compression, thereby exerting increased tension on the surrounding fibers. A new idea for treating cancer appears to weaken the connection between cells and the fibrous matrix [320]. With a more flexible cell-matrix interface, neither cancer cells nor tissue need to be softened, as it is assumed that a stiffer environment results in increased cell damage and mutations in cancer cells.

Upon mechanosensing and mechanotransduction Yap translocates into the nucleus of cancer cells and acts as a transcriptional activator that subsequently induces the expression of several genes [322]. The mechanisms, which comprise an impairment of cytoskeletal contractility [39,323,324,325], distortion of the F-actin cytoskeleton [324], and a reduction in F-actin capping proteins, considerably impact YAP function [325]. YAP is the principal effector of the Hippo-Warts kinase cascade in Drosophila melanogaster, where its ortholog is termed Yorkie (Yki). YAP/Yki acts as a transcription coactivator that is controlled through the Hippo-Warts kinase cascade, which phosphorylates and inactivates it to modulate cell growth, cell proliferation, and size of organs. When the Hippo-Warts signaling route is active, YAP/Yki stays in its inactive state. In this pathway, YAP is phosphorylated, which keeps the protein within the cytoplasm and inhibits its movement into the nucleus to activate expression of genes. YAP/Yki exists in its active state when the Hippo-Warts signaling route is inactive, ensuring that YAP is not phosphorylated and can relocate to the cell nucleus. When YAP/Yki enters the cell nucleus, it can bind to the transcription factor TEAD, known as Scalloped in Drosophila melanogaster, and activates the transcript of target genes implicated in proliferation and growth of cells.

In mammals, the mammalian sterile 20-like 1/2 (MST1/2) and large tumor suppressor kinase 1/2 (LATS1/2) negatively regulate YAP, which is then inactive [326,327]. Activated LATS1/2 subsequently phosphorylates the transcription coactivator YAP/TAZ and induces its attachment to 14-3-3 proteins, causing it to be trapped in the cytoplasm or broken down through the ubiquitination pathway. YAP/TAZ that does not pass into the cell nucleus cannot attach to the nuclear transcription factor TEAD1-4 [317]. α-catenin can control YAP/Yki, by several, possibly cell-type-specific ways, among them cytoplasmic sequestration or the activation of an integrin-Src signaling route [328,329,330]. In addition, active Yki not just functions in the cell nucleus promoting growth, but is also bound to apical junctions where it increased the activity of myosin II that in turn fosters the tissue growth via the Hippo/Yki signaling route [331]. When α-catenin ties up with the cadherin-β-catenin complex, its ability to tie up with actin is compromised, and in reverse. This dynamic switching, which is fueled by conformational alterations in α-catenin itself, enables it to act as a molecular switch that governs the actin cytoskeleton’s dynamics and assembly across cell–cell junctions. In conclusion, it can be said that mechanical memory can be stored within different molecules and different cellular structures. Moreover, the theory of the MeshCODE offers an original and fascinating prospect for accessing biological digital data. However, this theory is still in its early stages and requires some further refinement. For the sake of completeness only, at the level of mechanoreceptors, such as integrins, a hybrid theoretical model has been proposed [35]. The hybrid model combines a mechanical model and an Agent-Based Model (ABM) and it is hence referred to as Mech-ABM model.

## 7. Is There a Need to Define the Hypothesis of Cancer Memory in Terms of Mechanical Memory More Exactly?

**Figure 8:**
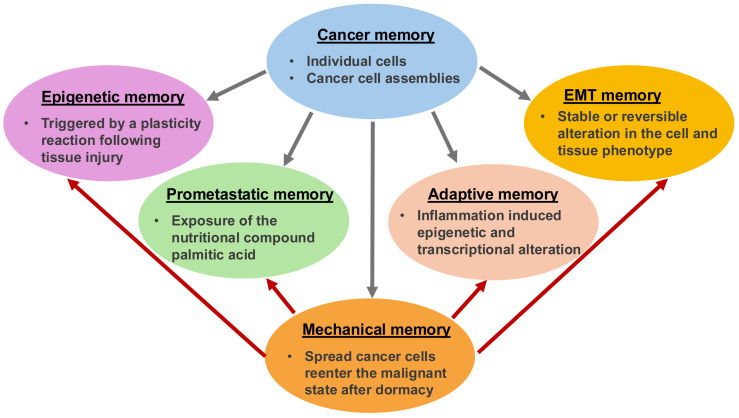
Cancer memory comprises different subtypes of memory, such as epigenetic memory, prometastatic memory, adaptive memory, EMT memory and mechanical memory, which is indicated by the grey arrows. The various subtypes of memory are not clearly separated from each other in terms of mechanical memory, as it is present in all types of memory and appears to play a decisive role, which is depicted by the red arrows.

Cancer memory has been proposed as a potential mechanism through which intricate knowledge, like metastatic phenotypes, resistance to cancer treatment, and patterns of engagement with the TME, could be codified at various levels through similar mechanisms that are involved in memory generation within the brain and immune system [332]. Among these mechanisms are epigenetic alterations in individual cells and scattered state alterations in assemblies of cells (Figure 8) [332].

It is known that cancer cells and their precursors store phenotypic conditions in epigenetic memory that codify malignancy in a manner not based on alterations in DNA sequence. Transient transcriptional modulation can cause epigenetic memory in cancer. For example, transient transcriptional knockdown of Polycomb proteins causes an irreversible epigenetic switch to a cancer cell phenotype in Drosophila in the absence of genomic driver mutations [333]. Melanoma cells exhibit long-term fluctuating gene expression profiles that code for therapy-resistant phenotypes through PI3K and TGF-beta signaling routes, and store these cell states in their memory, indicating that culture conditions play a role for selecting a suitable model for cancer, such as melanoma [334]. This plasticity permits melanoma cells to transition to more invasive, therapy-resistant phenotypes by modifying their signaling pathways to evade targeted therapies such as MAPK inhibitors. Research indicates that activation of the PI3K signaling pathway may confer a survival benefit and a route for resistance to targeted treatments. Likewise, although the TGF signaling pathway occasionally enhances cell death, it can also enhance resistance, especially when the mediators, like SMAD3, are upregulated. Activation of the PI3K-AKT signaling route can cause resistance to MAPK/ERK inhibitors, thus permitting cells to remain viable and render them less reliant on the MAPK signaling route. For instance, the inhibition of the MAPK signaling route can thereby be compensated. The involvement of the TGF signaling pathway is intricate. It may be associated with resistance, particularly at increased concentrations of its effector SMAD3 [335]. In certain situations, the TGF signaling route can also trigger cell death, although other mechanisms such as PI3K activation can antagonize its effects. The MAPK signaling route is a frequent target of treatments, but its blockage can result in the activation of other compensatory signaling routes, like PI3K, to encourage resistance.

The blocking of PI3K confers sensitivity to subsequent BRAF- and MEK-impairment treatments [334]. The epigenetic memory triggered by the plasticity reaction following tissue injury primes epithelial cells for consecutive carcinogenesis [336]. Following skin damage, remote epithelial stem cells engage an epigenetic memory mechanism that prepares them for increased wound healing in the future. This alteration in cell state promotes later cancer development [337]. Cancer cells likewise exhibit a metastatic latent period [338] during which spread cancer cells return to their malignant condition after prolonged quiescent phases as a potential memory retrieval mechanism. After being primed through the tissue surrounding them, cancer precursor cells keep their malignant traits in their epigenetic memory. Inflammation induces epigenetic and transcriptional alterations in pancreatic acinar cells, resulting in adaptive memory that primes these cells for consecutive acinar duct metaplasia and tumorigenesis a long time after the primary inflammation has dissipated [339]. Importantly, the transcription factor Egr1, a key memory regulator within the brain [340], plays a pivotal part in this mechanism. It has also been proposed that inflammation causes epigenetic memory in pancreatic acinar cells, which renders them susceptible to oncogenic transformation following a KRAS mutation [341]. Importantly, this memory can be counteracted through MAPK blockade, which raises the barrier for cancer transformation. Cancer cells can preserve a prometastatic memory even after receiving the nutrient compound palmitic acid, and this phenotype remains in place even once the stimulus is withdrawn [342]. A further memory mechanism in cancer is the emergence of a mechanical memory that controls the effectiveness of subsequent metastasis [285]. During this process, the engagement of cancer cells with the microenvironment of the primary solid tumor triggers a form of mechanical stimulus-dependent mechanical memory that fuels the malignant behavior throughout metastasis [285]. During the malignant progression of cancers, phenotypic alteration, such as transitions in cancer cells can occur. For example, epithelial-mesenchymal transition (EMT) and hybrid EMT or jamming, key processes in multiple types of cancer [343,344], entail the attainment and retention of stable alterations in cell and tissue properties and are therefore a memory process. Earlier studies have focused on EMT memory [257] and the activation of CREB, which is a memory controller in the brain [345] and the immune system [346], s implicated in this transition mechanism [347], thus forming an intriguing bridge between these two mechanisms. In a similar way, Netrin1, which is implicated in synaptic plasticity and memory in the brain [348], appears to be pivotal for EMT [349].

There are two interesting lines of experimental findings. Firstly, cancer cells and their precursors harbor epigenetic memory with intricate functional behavioral phenotypes. Secondly, cancer cells utilize learning and memory processes as cancer advances, which are also employed physiologically in the brain and immune system, and the employment of these processes is positively linked to the malignant nature of the cancer. This supports the hypothesis that cancer cells code and recall memories in a similar way to the brain and the immune system. Cancer memory can be divided into basal cancer memory and complex cancer memory. Malignant phenotypes can be codified at the cellular scale in the basal cancer memory, which has been identified in a number of current studies, such as NMDA receptors (NMDARs) which are involved in the positive regulation of the progression of breast cancer [350], pancreatic cancer [351] and melanoma [352]. There are similarities between cancer memory and the regulation of synaptic plasticity and memory formation in the brain [353] as well as neural survival [354], which occurs via NMDARs. NMDAs are also implicated in the regulatory function of the adaptive immune system [355,356]. Epigenetic memory codified in histone modifications and DNA methylation represents one of the most important mechanisms for storing cellular conditions in memory [357,358] and cancers can utilize these mechanisms to codify malignant phenotypes in response to various kinds of environmental stimuli. Moreover, as in the brain, in which specific cognitive representations are not fully traceable to the scale of individual cells for example through genome sequencing, cancer may store information at higher scales within a complex cancer memory. This phenomenon fits well with the observation that cancers can build chemically and electrically connected networks of cells with diverse functional structures. Perhaps cancer codify phenotypes in multicellular groups, creating so-called cancer memory engrams.

By utilizing physiological multiscale learning mechanisms, cancer cells could work out how to persist and propagate within the host organism. Recent work has in fact described cancer propagation in terms of a learning mechanism [359]. In the cancer memory model cancer gains the capacity to act on its own by disconnecting itself from the remainder of the organism and coding “cancer memories” for the purpose of ensuring its survival. The coding can occur at the level of single proteins, such as the focal adhesion proteins talin and vinculin or at the level of multicellular assemblies, such as a collection of cancer cells. By creating and retrieving its own memories at the cost of the entire organism, cancer arises as an individual inside the organism in a process that can be termed oncogenic individuation. A key problem then is how individual cells come to realize that they are members of this newly formed entity. It is possible that specific stably inherited hallmarks, like DNA mutations and epigenetic modifications, have the role of labeling and communicating a cell’s membership in the cancerous individual as distinct from the remainder of the host body. Some selected cancer hallmarks could represent a fundamental programming defect, causing cancers to revert to a selfish mode of operation. Earlier work has revealed that gene expression profiles can be robustly passed down through various clones of the same mammalian cell type [360], which offers evidence that epigenetic memory can be conferred through cell generations. Cells that are linked to one another by these defined hallmarks may use learning and memory mechanisms to retain beneficial information that assists them in adapting to the numerous demands of oncogenic advancement. Since memory generation constitutes a pivotal mechanism in the linkage of individual active substances [361], the development of cancer memories could represent the foundation for the individuation mechanism of the “suborganism”, referred to as oncogenesis.

Oncogenesis appears to be a maladaptive mechanism that shares functional similarities with apparently unconnected abnormalities in other learning processes, such as post-traumatic stress disorder (PTSD) and addiction in the brain. In both of these latter diseases, environmental stress causes a malignant reprogramming of a learning pathway, such as the memory loops in the brain, with consequent disconnection of regulatory cues, hyperoptimization of the system’s local goal attainment, and ongoing deterioration at the expense of the entire organism. There exist not merely functional similarities, but also numerous molecular mechanisms, such as adaptive transcription, that play a role in oncogenesis and the development of psychiatric disorders [362].

The hypothesis of cancer memory is capable of coexisting to different extents with theories of clonal evolution. It has been shown that memory coding in the brain entails intercellular concurrence driven by single-cell characteristics [363]. For instance, neurons with enhanced intrinsic excitability are favorably committed to memory engrams [364], indicating that evolutionary cell population dynamics may contribute as underpinning supports for memory formation. In a similar way, antigen-specific reaction profiles in the immune system comprise the clonal selection of a specific subtype of lymphocytes [365], which subsequently establishes an immune memory. There are pronounced resemblances between the brain and cancer in terms of clonal evolution on the basis of genomic diversity, as neurons and cancer cells exhibit genomic diversity, such as physiological differences in the karyotype of neurons [366] and in the sequence of the genome [367], deviations in the karyotype of cancer cells [368] and the sequence of the genome [369]. Genome mutations may be used by both learning systems, the brain and cancer, to improve responsiveness and therefore adaptability. An intriguing aspect is that the underlying cause-and-effect connection of genomic abnormalities and cancer development contradicts the conventional understanding that genomic typically alterations lead to cancer. Alternatively, cancer may initially emerge as a higher-order unit, perhaps in several cases, and subsequently utilize genomic divergence via elevated mutation frequencies and genomic reassortments to enhance its reaction diversity for learning objectives. Clonal evolution may also involve cells developing a memory in response to a specific cue, thereby serving as a facilitator of cancer memory. It is well established that clones with superior learning and multicellularization abilities improve their survivability against cancer progression. Cancer memory theory seems to be consistent with models of clonal evolution that include epigenetic changes as heritable [370]. The cancer memory hypothesis is at odds with models that propose clonal evolution as the solely responsible reason for cancer, on the basis of random genetic mutations. Cancer memory posits that the flexible coding, retrieval, and alteration of memories are the key contributors to malignancy. A model that therefore links clonal evolution with cancer memory would assume that clonal evolution improves the memory-forming abilities of cancers and thus promotes cancerous growth and metastasis. However, there is a need to incorporate mechanical memory into the theory of cancer memory, as it is also present at both the cellular and tissue levels, where cancer memory also occurs. Mechanical memory can apparently influence other types of memory, such as epigenetic, prometastatic, EMT, and adaptive memory, which play crucial roles in cancer memory. Therefore, it can be proposed that cancer memory also includes mechanical memory in all the different stages of cancer. Consequently, the theory of cancer memory is important, but requires refinement in terms of the involvement of mechanical memory.

## 8. Conclusions and Future Directions

Cancers can be considered biological learning and memory agents, as they leverage the potency of complex learning mechanisms for their own survival while not engaging with the overarching functional rationale of the physiological processes of the entire metazoan body. Cancer can store malignant phenotypes within epigenetic memory and, as cancer progresses, utilize various mechanisms that deploy learning and memory mechanisms similar as in the brain and immune system. Cancer cells could therefore generate, memorize, and recall intricate memories for survival and more efficient propagation during metastasis. Oncogenesis appears to develop as a process of individuation inside the organism, where the components of cancer are linked by the creation of cancer memories. The hunt for new ways cancer spreads could be guided by the way the brain and immune system use molecular and cellular mechanisms to code high-level knowledge into memories. Gaining an in-depth insight into the cancer memory code could facilitate the development of novel treatment approaches, including cancer memory erasure concepts and novel drug treatments that disrupt cancer memory programs. This phenomenon has profound clinical consequences in fields like stem cell therapy, tissue engineering, and metastatic spread of cancers as it can impact cell plasticity, treatment efficacy, and overall progress of the disease. Developing a deeper comprehension of and managing mechanical memory through approaches such as utilizing soft substrates or working with epigenetic modifications can contribute to enhancing cell-based approaches and improving the treatment of disorders such as cancer. The mechanism of mechanical memory involves the reorganization of the cytoskeleton, epigenetic alterations and the activation of transcription factors that translocate into the nucleus. For the purpose of the redesign of the cytoskeleton, cells can alter their own internal cytoskeleton, especially the actin filaments, in response to mechanical stimuli such as stiffness. This results in a memory of the previous mechanical setting. Besides cytoskeletal changes, there are epigenetic modifications caused by long-term mechanical effects, which alter chromatin structure and gene expression and can thus further consolidate mechanical memory. Another further effect of the mechanical memory process is the activation of transcription factors. In this process, pivotal transcription factors like YAP react to mechanical cues and convert physical signals into alterations in gene expression, thereby ensuring a persistent cellular mechanophenotype. When fighting cancer, several clinical implications need to be taken into account. For example, in cancer metastasis, mechanical memory allows cancer cells to preserve adaptive characteristics from their high-strength primary TME, improving their capacity to both survive and colonize in more distant tissues. When stem cell therapies and tissue engineering are employed to treat cancer, it needs to be recognized that cells seeded and cultured on rigid plastic products can gain a mechanical memory that negatively influences their ability to adequately differentiate into soft, native tissue following engraftment. In a similar manner, when treating wound-healing disorders associated with cancer, the rate and efficacy of wound repair can be influenced via the stiffness of the epithelial cells, whereby continuing high contractility and migration velocity are maintained even after transition to softer surroundings. There are several strategies for regulating mechanical memory in cells, which either affect the extracellular environment of the cells or directly impact the cells themselves. Three of them are outlined below. The first strategy is to exploit soft substrates. The growth of cells on softer substrates can contribute to attenuating the negative impacts of mechanical memory and encouraging more pleasing cellular phenotypes. Moreover, the realization that cancerous tissue has a tendency to be stiffer compared to healthy tissue has resulted in the establishment of lysyl oxidase inhibitors as anti-stromal therapies that aim to disrupt the crosslinking of the ECM to subsequently soften it [371]. The second strategy is the epigenetic manipulation approach. Blocking specific enzymes implicated in epigenetic reorganization may possibly delete or revert mechanical memory, thus enabling cells to more easily adjust to new mechanical stimuli. The third strategy is the controlled mechanical loading approach. The utilization of dynamic substrates or defined mechanical loading during the culture procedure may also be employed to shape or alter the mechanical memory for several therapeutic or experimental applications.

An evolving important new concept is that of mechanical memory, whereby cells maintain a memory of their engagement with a prior mechanical stimulus. Nevertheless, the magnitude of this memory is affected by the strength and length of a mechanical stress that can cause the cell to undergo lasting epigenetic alterations [189]. These alterations can be described as a reprogramming of the cell. Additional investigations are required to ascertain whether the mechanical memory acquired during biophysical adjustments in a primary TME prepares cells to withstand and survive a secondary TME [285]. The development of therapeutic blockers of epigenetic modifiers to perturb mechanical memory could possibly contribute to the invention of a new class of therapeutics against metastasis. In general, several conclusions can be drawn. Mechanical memory is a universal phenomenon among cancers. It is not just cancer cells but also other cells like endothelial cells that exhibit mechanical memory. Mechanical memory regulation and storage can take place at different levels, such as chromatin, molecules, cell surface receptors, cytoskeletal structures and cell assemblies, such as tissues and organs. The ECM microenvironment can regulate mechanical memory and hence offers a therapeutic option for cancer metastatic spread. The MeshCODE theory bears similarities to the signal transduction processes of the nervous system, but probably also plays a role in cancer. However, additional investigations are required to uncover the precise mechanisms involved in mechanical memory acquirement and storage. Moreover, there are several implications of cellular mechanical memory in the field of bioengineering, where new models are urgently needed for the development of new therapeutic approaches for cancer metastasis [20].

## Figures and Tables

**Table 1:** Brief overview on selected important findings in mechanical memory identification in various (cancer) cell types.

Cancer Type/Cell Line	Mechanical Stimuli	Molecule/Pathway (for Mechanical Memory Storage)	Metastatic Site	Description/Reference(s)
Breast cancer	Fibrotic-like matrix stiffness	RUNX2	Bone marrow	[190]
Breast cancer and vulvar cancer (human mammary nontumorigenic MCF-10A cells, human mammary tumorigenic MCF-7 and human epidermoid carcinoma A431)	Increased stiffness of PDMS hydrogels	YAP activity (stores mechanical memory)		Regulation of collective cell migration via mechanical memory of past substrate stiffness in a 2D migration assay [191]
Breast cancer (MDA-MB-231)	Stiffness (and topology)	Elevated LINC expression, integrins, FAK and vimentin	Lung	Regulation of motility and sphere formation in 3D porous PCL scaffolds; regulation of metastatic spread in vivo (mice) [192]
Breast cancer (SUM149 and patient derived cells Vari-068 cells)	Stiffness (stiff 28 kPa and soft 1.5 kPa)	Elevated basal activation of Akt and ERK		Signaling is transmitted via two major receptors, CXCR4 and EGFR. Enhances signaling via Akt and ERK kinases through activation through the ligands CXCL12-α and EGF, respectively that induces migration of triple negative breast cancer (TNBC) cells [193].
Breast cancer (MDA-MB-231 (metastatic breast cancer), HT1080 (fibrosarcoma), and HFF-1 (fibroblasts) cells)	Polyacrylamide hydrogels that mimic soft (approx. 1 kPa) and stiff (approx. 34 kPa) ECM scaffolds on which the cells are cultured for three or seven days	The transcription factor NFATC2’s is not present in metastatic cells and is identified as a key mechanical memory mediator	Lung	NFATC2’s function in mechanotransduction is unclear, but it is a likely regulator of the mechanical memory phenotypes observed. Its stiffness-induced expression, which is lacking in metastatic cells, implies that fibroblasts and fibrosarcoma cells utilize NFATC2 to preserve mechanical memory, whereas metastatic MDA-MB-231 cells circumvent this control to keep their plasticity intact within heterogeneous surroundings [194].
Pancreas ductal adenocarcinoma (PDAC)	Acute, short-term priming were performed in which PDAC cells are either grown on a soft (1 kPa) or stiff (10 kPa) environment for 6 h, then seeded into a new soft or stiff 3D surrounding for 18 h to carry out RNA-seq analyses for quantification of gene expression.	Tumor suppressors (LATS1, BCAR3, CDKN2C) and cancer-associated genes (RAC3)	Liver	The stiff 3D culture is linked to a downregulation of tumor suppressors, such as LATS1, BCAR3, CDKN2C and upregulation of cancer-associated genes, such as RAC3. Immunofluorescence staining of BCAR3 and RAC3 confirmed the persistence of this cellular response, with BCAR3 being upregulated in soft and RAC3 in stiff cultures [195].
Pancreatic cancer (SUIT-2.28)	Priming with soft or stiff environments	Mechanical memory relies on YAP activity	Liver	Epithelial cells prepared on a stiff matrix migrate more rapidly, exhibit higher actomyosin expression, develop larger focal adhesions, and keep their nuclear YAP after receiving on a soft secondary matrix, compared to their control response on a uniformly soft matrix. The priming effect on a soft ECM is the reverse. The mitigation of YAP greatly decreases this memory-dependent migration. Consequently, softly primed cells exhibited reduced YAP nuclear translocation and a reduction in YAP-driven stiffness perception [191].
Oral squamous cell carcinoma (OSCC); invasive SSC25 mesenchymal cells overexpress myosin II (vs. noninvasive Cal27 epithelial cells) consistent with invasive OSCC	Cell contractility underpins mechanical memory generation; cultured on soft 0.5 kPa or stiff 20 kPa matrices	The acquisition of the mesenchymal phenotype conferred through stiffness necessitated AKT signaling route and was also evident in patient specimens, while the reinstatement of the phenotype on soft substrates necessitated focal adhesion kinase (FAK) activity.	Lung, bone and liver	Extended exposure of Cal27 cells to a stiff niche or contractile agonists resulted in increased expression of myosin and EMT markers, permitting them to undergo migration as fast as SCC25 cells, which continued even after the niche softened, indicating a mechanical memory effect of their previous niche [196].

**Table 2:** The stiffness of tumor tissue is generally increased compared to healthy tissue in various cancer types. However, there is an exception in glioblastoma. Moreover, the stiffness of the tumor tissue rises due to the grade of malignancy of the cancer. The individual values vary between studies depending on the experimental preparation of the samples and/or very different biophysical analysis techniques that cannot be directly compared with each other.

Tissue	Normal Tissue Stiffness (kPA)	Tumor Tissue Stiffness (kPA)	Biophysical Technique	Reference
Breast/ mammary tissue	3.3	Low grade invasive ductal carcinoma (IDC): 10.4 DCIS: 16.4 High grade IDC: 42.5	-	[202]
Breast/ mammary tissue	Unimodal distribution: 1.1 ± 0.3	Invasive ductal carcinoma with bimodal stiffness distribution: 0.4 ± 0.3 (soft peak) 1.9 ± 0.6 (stiff peak)	AFM	[203]
Breast/ mammary tissue	0.2 ± 0.03	4.0 ± 0.9	Electromechanical computer-controlled indenter with a miniature linear stepper motor (minimal displacement 0.0032 mm), force transducer (load capacity 1.47 N), and a linear variable displacement transducer	[204]
Bladder	About 3	Recurrent cancer: about 13 Newly diagnosed cancer: about 8	AFM	[205]
Brain	Gliotic tissue (non-tumor gliosis): 0.01–1.8	0.2 (with soft ECM regions) 0.04–1.4 (lower grade gliomas (LGG)) 0.07–1.4 (glioblastomas (GBM))	AFM	[206]
Colorectum	0.9	Primary tumor stage: T1: 2.8 T2: 3.5 T3: 8.8 T4: 13.8 Distant metastasis: Present: 13.8 Absent: 7	Venustron system based elastography	[207]
Glioblastoma	1.5 ± 0.3 kPa	1.3 ± 0.3 kPa Glioblastoma	Magnet resonance elastography (MRE)	[208]
Liver	-	HCC: 55 CCC: 75 Metastatic cancer: 68.5	Transient elastography	[209]
Liver	<6	Disease state, such as fibrosis and cirrhosis: 8–12	FibroScan^®^ or vibration-controlled transient elastography (VCTE^TM^)	[210]
Liver	5.5 ± 0.9	Fibrotic HCC with medium lung metastatic capacity: 9.4 ± 0.5 Cirrhotic HCC with high lung metastatic capacity: 16.1 ± 1.0	Laser scanning microscope using FibroScan^®^ (elastic wave generation via a vibrator)	[211]
Liver	-	Low level of malignancy: 8–15 High level of malignancy: 14–18	AFM	[212]
Liver	1.5–5	-	Shear elasticity	[213]
Lung	2.0 ± 0.1	Idiopathic pulmonary fibrosis: 16.5 ± 2.3	AFM	[214,215]
Ovary	-	Mesenchymal high-grade serous ovarian cancers (HGSOC): 0–40 (soft) 0–120 (stiff) Non-mesenchymal less aggressive HGSOC: 0–40	Shear wave elastography (SWE)	[216]
Pancreas	0.4	1.2	AFM	[217]
Pancreas	1.2 ± 0.2 at 40 Hz 2.1 ± 0.3 at 60 Hz and 2.5 ± 0.1	6.1 ± 0.5	1.5-T and 3-T MRE with an accelerated echo planar imaging (EPI) pulse sequence with low-frequency vibrations (40 and 60 Hz)	[218,219]
Pancreas	<15	>40	Harmonic motion elastography (HME)	[220]

## Data Availability

No new data were created or analyzed in this study.

## Abbreviations

The following abbreviations are used in this manuscript:

ABD	Actin binding domain
AFM	Atomic force microscopy
BCAR1	Breast cancer anti-estrogen resistance 1
CAFs	Cancer associated fibroblasts
CTCs	Circulating tumor cells
ECM	Extracellular matrix
EDA-FN	Extradomain A fibronectin
EMP	Epithelial to mesenchymal plasticity
GEF	Guanine nucleotide exchange factor
GPCRs	G protein coupled receptors
HATs	Histone acetyltransferases
HDACs	Histone acetyllases
hMSCs	Human mesenchymal stem cells
IDC	Invasive ductal carcinoma
IFP	Interstitial fluid pressure
LATS1/2	Large tumor suppressor kinase 1/2
MST1/2	Mammalian sterile 20-like 1/2
MeCo	Mechanical conditioning
MET	Mesenchymal to epithelial transition
MMP	Matrix-metalloproteinase
MOBKL1A/B	MOB kinase activator 1A/B
MRE	Magnet resonance elastography
NMDAs	NMDA receptors
PDAC	Pancreas ductal adenocarcinoma
PDMS	Polydimetylsiloane
PTSD	Post-traumatic stress disorder
RIAM	Rap1-GTP-interacting adaptor molecule
SFKs	Src-family tyrosine kinases
α-SMA	Alpha-smooth muscle actin
TAMs	Tumor associated macrophages
TAZ	Transcriptional regulator
TEAD	Transcription enhancer of activators domain
TME	Tumor microenvironment
TRPC1	Transient receptor potential cation channel subfamily C member 1
TRPM7	Transient receptor potential cation channel subfamily M member 7
TRPV4	Transient receptor potential cation channel subfamily V member 4
VBSs	Vinculin binding sites
VEGF	Vascular endothelial growth factor
YAP/TAZ	Yes-associated protein/transcriptional co-activator with PDZ-binding motif
Yki	Yorkie
